# A dedicated diribonuclease resolves a key bottleneck for the terminal step of RNA degradation

**DOI:** 10.7554/eLife.46313

**Published:** 2019-06-21

**Authors:** Soo-Kyoung Kim, Justin D Lormand, Cordelia A Weiss, Karin A Eger, Husan Turdiev, Asan Turdiev, Wade C Winkler, Holger Sondermann, Vincent T Lee

**Affiliations:** 1 Department of Cell Biology and Molecular Genetics University of Maryland College Park United States; 2 Department of Molecular Medicine, College of Veterinary Medicine Cornell University Ithaca United States; Rutgers University United States; National Institute of Child Health and Human Development United States

**Keywords:** *Pseudomonas aeruginosa*, *Vibrio cholerae*, oligoribonuclease, diribonucleotides, diribonuclease, Other

## Abstract

Degradation of RNA polymers, an ubiquitous process in all cells, is catalyzed by specific subsets of endo- and exoribonucleases that together recycle RNA fragments into nucleotide monophosphate. In γ-proteobacteria, 3-‘5’ exoribonucleases comprise up to eight distinct enzymes. Among them, Oligoribonuclease (Orn) is unique as its activity is required for clearing short RNA fragments, which is important for cellular fitness. However, the molecular basis of Orn’s unique cellular function remained unclear. Here, we show that Orn exhibits exquisite substrate preference for diribonucleotides. Crystal structures of substrate-bound Orn reveal an active site optimized for diribonucleotides. While other cellular RNases process oligoribonucleotides down to diribonucleotide entities, Orn is the one and only diribonuclease that completes the terminal step of RNA degradation. Together, our studies indicate RNA degradation as a step-wise process with a dedicated enzyme for the clearance of a specific intermediate pool, diribonucleotides, that affects cellular physiology and viability.

## Introduction

Degradation of RNA is initiated by endonuclease-catalyzed cleavages; the resulting oligoribonucleotide fragments are hydrolyzed to completion by a mixture of exoribonucleases for the maintenance of cellular nucleotide pools (Hui et al., 2014). Unlike the conserved machineries for the synthesis of macromolecules, distinct sets of RNases are used by different organisms to degrade these oligoribonucleotides. *Escherichia coli* genomes encode eight 3’−5’ exoribonucleases, namely polynucleotide phosphorylase, RNase II, D, BN, T, PH, R and oligoribonuclease (Orn) (Hui et al., 2014; Bandyra and Luisi, 2013). A subset of these enzymes recognizes structural features of the RNA substrate, such as in tRNA, while others act on unstructured polymers (Li and Deutscher, 1996).

As an exoribonuclease, Orn is unique for two reasons. First, *orn* is required for viability in many γ-proteobacteria, including *E. coli* (Ghosh and Deutscher, 1999) and other organisms (Palace et al., 2014; Kamp et al., 2013), unlike all other known 3’−5’ exoribonucleases. This indicates that not all exoribonucleases have redundant functions despite acting on the 3’ end of RNA substrates and in several cases, including RNase R and RNase II, sharing an activity toward oligoribonucleotides (Zuo and Deutscher, 2002; Cheng and Deutscher, 2002; Cheng and Deutscher, 2005; Frazão et al., 2006). Hence, Orn appears to catalyze a particularly important step in RNA turnover. Second, Orn is a key enzyme in bacterial cyclic-di-GMP (c-di-GMP) signaling. The nucleotide second messenger c-di-GMP is produced in bacteria in response to environmental cues and controls a wide range of cellular pathways, including cell adhesion, biofilm formation, and virulence (Krasteva and Sondermann, 2017; Römling et al., 2013; Hengge, 2009; Jenal et al., 2017; Hall and Lee, 2018). Since the discovery of c-di-GMP over 30 years ago, it has been known that the signal is degraded by a two-step process with a linear pGpG diribonucleotide intermediate (Ross et al., 1987). While the enzyme for linearizing c-di-GMP to pGpG was discovered early on (Tal et al., 1998; Galperin et al., 1999), the identity of the enzyme that degrades pGpG remained elusive. Two recent studies showed that Orn is the primary enzyme that degrades pGpG in *Pseudomonas aeruginosa* (Cohen et al., 2015; Orr et al., 2015). In an *orn* deletion strain, the accrual of linear pGpG has a profound effect on cells. Specifically, the increase in pGpG inhibits upstream phosphodiesterases that degrade c-di-GMP, thereby triggering phenotypes associated with high cellular c-di-GMP levels. However, the molecular basis of Orn’s unique cellular functions in **γ**-proteobacteria that distinguishes it from all other exoribonucleases remains unexplained.

Since its discovery over 50 years ago (Stevens and Niyogi, 1967), Orn has been presumed to degrade oligoribonucleotides (Niyogi and Datta, 1975; Datta and Niyogi, 1975; Yu and Deutscher, 1995). This notion largely derived from assays utilizing two types of substrates. In one series of experiments, ^3^H polyuridine (poly(U)) was incubated with Orn, other enzymes, or lysates and analyzed by paper chromatography, which offers limited resolution overall (Niyogi and Datta, 1975; Datta and Niyogi, 1975; Yu and Deutscher, 1995). In a second set of experiments, Orn was incubated with oligoribonucleotides that had been tagged at their 5’ terminus by a large fluorophore. The products of this reaction were resolved by denaturing polyacrylamide gel electrophoresis, thereby allowing for detection of products with one or more nucleotides removed from the 3’ terminus (Cohen et al., 2015; Mechold et al., 2006; Mechold et al., 2007; Fang et al., 2009; Liu et al., 2012). In both instances, it was concluded that Orn could processively degrade ‘short’ oligoribonucleotides. Yet, it was not clear how Orn might selectively target ‘short’ RNAs, rather than simply binding to the penultimate sequence at the 3’ termini of single-stranded RNA of any length.

Here, we sought to rigorously examine Orn’s substrate preferences, revealing its unique properties. To that end, we incubated Orn with 5’ ^32^P end-labeled RNAs of varying lengths and analyzed the products of that reaction over time. Our data show that Orn exhibits a surprisingly narrow substrate preference for diribonucleotides. This finding is in stark contrast to the previous studies that described a broader substrate length range and that originally gave oligoribonuclease its name. We sought to understand this remarkable substrate selectivity of Orn by determining the crystal structures of Orn in complex with pGpG and other linear diribonucleotides. These data reveal a structural basis for the diribonucleotide preference and identify key residues for recognizing diribonucleotides. Furthermore, we find that Orn is the only diribonuclease in *P. aeruginosa*. Additionally, we show that other diribonucleotides, in addition to pGpG, can affect bacterial physiology. From this we propose a general model of RNA degradation, wherein a combination of exoribonucleases process oligoribonucleotides down to diribonucleotides and Orn completes RNA recycling by cleaving diribonucleotides to nucleoside monophosphates. In this way, Orn occupies an important but discrete step in the overall RNA degradation pathway.

## Results and discussion

### Orn functions as a diribonuclease in vitro

To understand the length preference of Orn, recombinant affinity-tagged *Vibrio cholerae* Orn (Orn*_Vc_*) was purified and tested biochemically. We used an established ligand-binding assay (Roelofs et al., 2011) to determine the relative substrate affinities of Orn to 5'-radiolabeled oligoribonucleotides that ranged from two to seven nucleotides in length. Unlike fluorophore-labeled RNA used in several earlier studies (Cohen et al., 2015; Mechold et al., 2006; Mechold et al., 2007; Fang et al., 2009; Liu et al., 2012), radiolabeling with ^32^P ensures that the substrate structure is unperturbed compared to native ligands. The assays were also performed in buffer lacking divalent cations to prevent complication due to enzyme activity. When spotted on nitrocellulose membranes, protein and protein-substrate complexes are sequestered at the point of application to the membrane, whereas unbound substrate diffuses due to radial capillary action (Roelofs et al., 2011). By this assay, quantification of the diffusion zone reveals that Orn*_Vc_* exhibits the highest affinity for diribonucleotide (*K_d_* ^pGpG^ = 90 -/+9 nM), as compared to oligoribonucleotides of greater lengths (Table 1) (Orr et al., 2015). The observed affinity of Orn*_Vc_* with the purification tag removed (*K_d_* ^pGpG^ = 80 -/+9 nM) was similar indicating the tags did not alter the interactions (Figure 3—figure supplement 1). Increase in the length to three or four residues reduces the affinity 7- or 10-fold, respectively (Table 1). Substrates with five or more bases show a greater than 28-fold reduction in affinity compared to diribonucleotides. These results suggest that Orn has a strong preference for diribonucleotides over longer oligonucleotides.

**Table 1.** Quantitative measurement of length-dependent oligoribonucleotide affinities.

Substrate^a^	Dissociation constant *K_d_* (nM)^b^
GG	90 -/+9
AGG	630 -/+80
AAGG	890 -/+90
AAAGG	2,560 -/+170
AAAAGG	3,830 -/+370
AAAAAGG	3,750 -/+530

^a^: RNAs were labeled with ^32^P at their 5’ end. ^b^: Affinities were measured using an established binding assay for radiolabeled RNAs (Roelofs et al., 2011; Patel et al., 2014).

**Figure 1.**
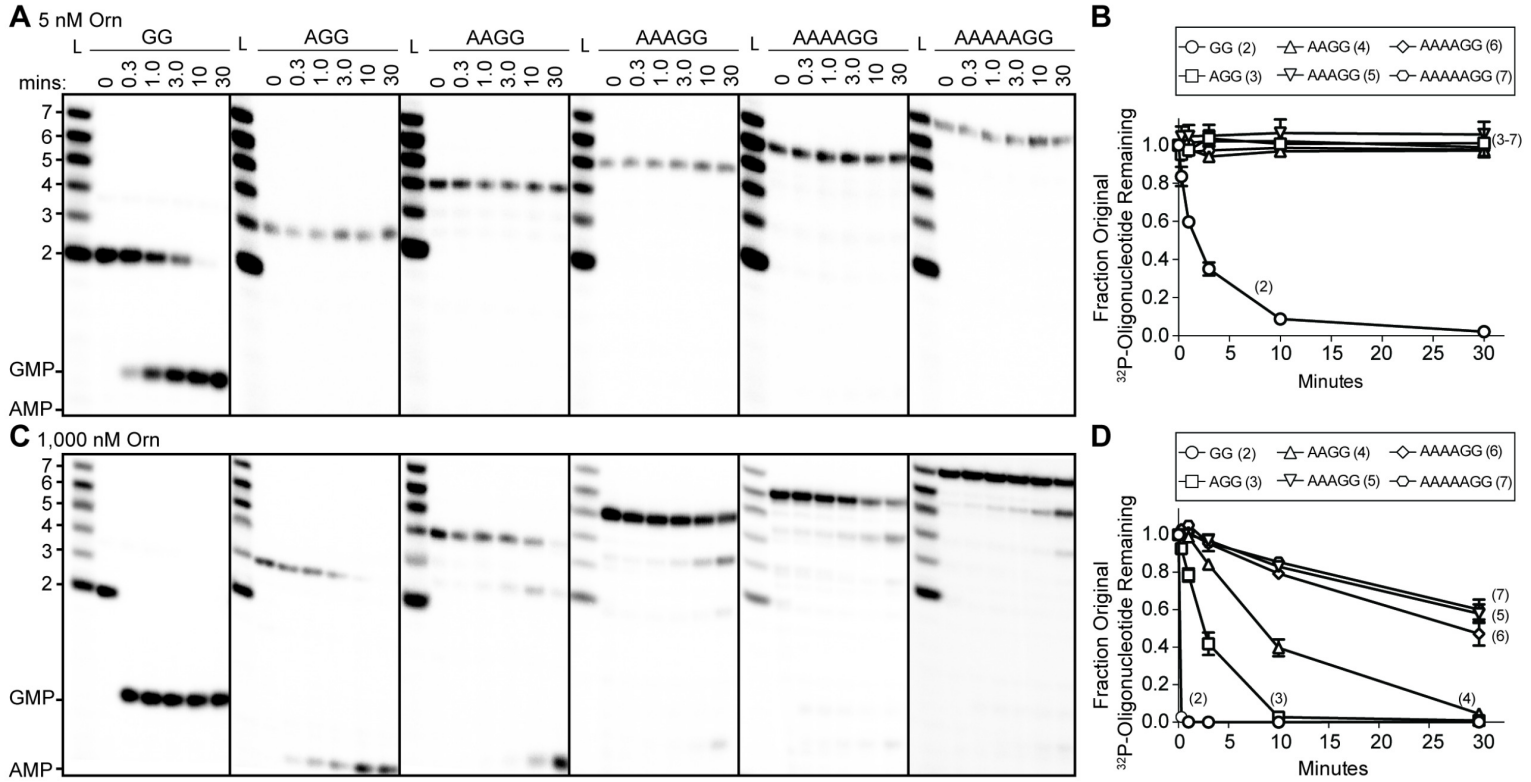
Orn has a stark preference for diribonucleotide cleavage in vitro. RNA nucleotides two to seven residues in length (1 μM, containing the corresponding ^32^P-labeled RNA tracer) were each subjected to cleavage over time with 5 nM (**A, B**) or 1000 nM Orn*_Vc_* (**C, D**). Aliquots of each reaction were stopped at indicated times (min), and assessed by denaturing 20% PAGE (**A, C**). Quantification of the intensities of bands corresponding to the amount of uncleaved initial oligonucleotide over time are plotted as the average and SD of three independent experiments (**B, D**). Figure 1—source data 1.Numerical data for Figure 1B and D.

To understand whether the affinity preference reflects nuclease activity with natural substrates that are unmodified at the 5’ end, we incubated Orn*_Vc_* with 5'-^32^P-radiolabeled oligoribonucleotides of varying lengths in the presence of divalent cations that support catalysis. The products of these reactions were resolved by urea-denaturing 20% polyacrylamide gel electrophoresis (PAGE). Under these electrophoresis conditions, the mononucleotides and oligonucleotides that were tested (between 2 and 7 nucleotides in length) can be resolved. The diribonucleotide substrate in this experiment was pGpG (GG), whereas the longer oligoribonucleotides included an increasing number of adenine nucleotides at the 5’ end. This arrangement ensured that the same GG sequence was maintained at the 3’ end while also avoiding stable G quadruplex formation that may be observed with RNAs containing stretches of poly-G (Kwok and Merrick, 2017). At substrate concentrations that far exceed enzyme concentration (200:1), the diribonucleotide substrate was already fully processed to nucleoside monophosphates by 30 min (Figure 1A) (Orr et al., 2015). In contrast, longer substrates (i.e. from 3-mers to 7-mers) were not processed at their 3' end, even at 30 min (Figure 1A and B). These results indicate that the length preference for exoribonuclease activity of Orn on diribonucleotides is even greater than mere differences in binding affinities. This strong substrate preference stands in stark contrast to previous studies arguing Orn acts as a general exoribonuclease that cleaves oligoribonucleotides with two to seven residues in length (Cohen et al., 2015; Niyogi and Datta, 1975; Datta and Niyogi, 1975; Mechold et al., 2006; Mechold et al., 2007).

**Figure 1—figure supplement 1.**
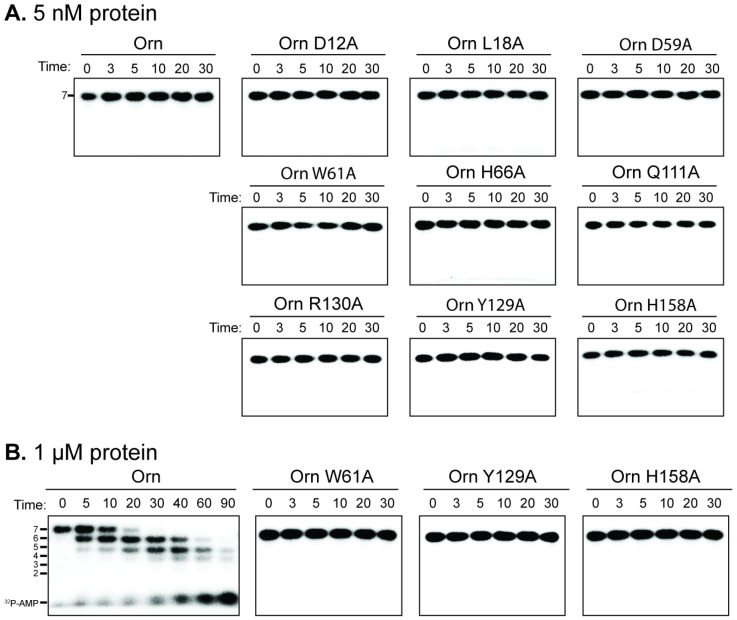
Catalytic residues of Orn*_Vc_* are required for degradation for ^32^P-AAAAAGG in vitro. Degradation of ^32^P-AAAAAGG by purified indicated His_10_-MBP-Orn*_Vc_* variants with 5 nM (**A**) or 1 µM (**B**). Samples were stopped at indicated time (min) and analyzed by denaturing 20% PAGE. Representative gel images of two independent assays are shown.

To determine if Orn can indeed cleave substrates longer than a diribonucleotide, the enzyme was incubated with RNA substrates at a 1:1 molar ratio. Under these conditions, the diribonucleotide substrate was completely processed to nucleoside monophosphates by the earliest time point, 20 s (Figure 1C and D). Orn*_Vc_* also facilitated the degradation of the longer RNA substrates, but only after significantly longer incubation times. For example, it required 10 min and 30 min to fully degrade 3-mer and 4-mer RNAs, respectively (Figure 1C and D). Cleavage was reduced further for longer RNAs; only 40–53% of the 5-mer, 6-mer and 7-mer RNAs were processed to nucleoside monophosphates at 30 min (Figure 1C and D), correlating with the weak affinities determined for these substrates (Table 1). Nonetheless, cleavage of the 7-mer required enzymatic activity since catalytically inactive Orn variants were unable to degrade the 7-mer (Figure 1—figure supplement 1). Of note, we observed a non-uniform distribution of degradation products for the longer RNA substrates. Specifically, the diribonucleotide intermediate was never observed as a reaction intermediate for the longer RNAs. This reaction pattern indicates that RNAs of more than two residues could accumulate, but that diribonucleotide RNAs were always rapidly processed to nucleoside monophosphates. Together, these results indicate that Orn exhibits a strong substrate preference for diribonucleotides over longer oligoribonucleotides – far greater than reported previously (Cohen et al., 2015; Mechold et al., 2006). While assays here were performed at near-physiological ionic strength, prior studies often utilized buffer lacking salt (NaCl/KCl), which may at least in part contribute to the apparent differences in catalytic activity (Niyogi and Datta, 1975; Ghosh and Deutscher, 1999; Cohen et al., 2015; Mechold et al., 2006).

### Structure of Orn complexes with pGpG and other linear diribonucleotides

**Figure 2.**
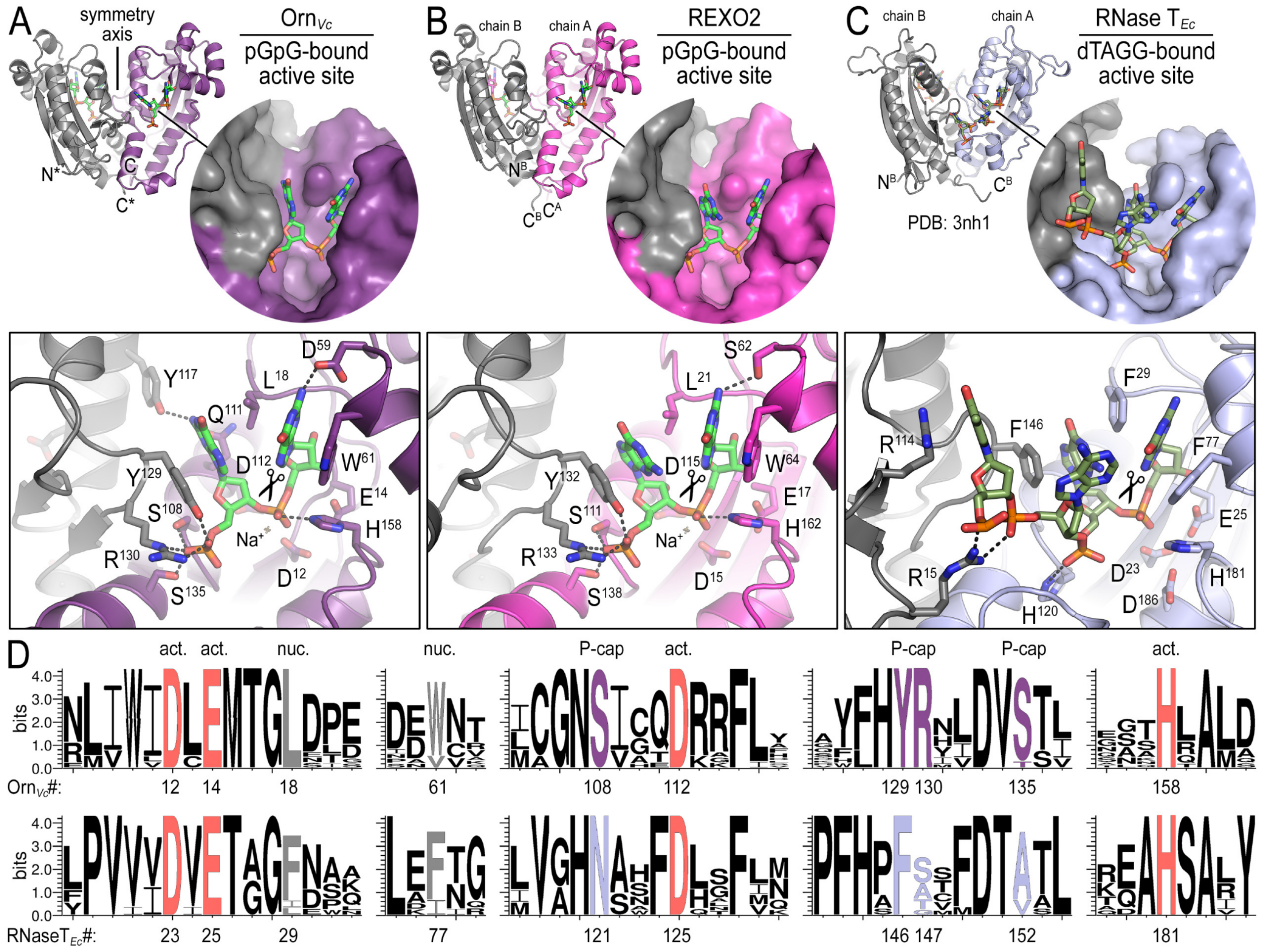
Structures reveal Orn’s conserved substrate preference for diribonucleotides. Crystal structures of pGpG-bound *V. cholerae* Orn (**A**) and human REXO2 (**B**) are shown in comparison to *E. coli* RNase T bound to substrate (PDB 3nh1; Hsiao et al., 2011) (**C**), another DnaQ-fold 3’−5’ exoribonuclease with a DEDD(h) active site motif. The top panels show ribbon representations of the dimeric enzymes. The insets are surface representations of the enzymes’ active sites shown in similar orientations. The bottom panel describes the active site residues involved in RNA binding and catalysis. Residue numbering for REXO2 refers to its cytosolic isoform lacking the mitochondria-targeting pre-sequence. The sequence logos in (**D**) were constructed based on multi-sequence alignments of Orn and RNase T orthologs. Overall sequence identity ranges from 43 to 70% for Orn and 46 to 69% for RNase T. Sequence identifiers are provided in Figure 2—source data 2. Sequence logos were plotted using WebLogo (Crooks et al., 2004). Conserved residues of the active site’s DEDD motif (‘act.’; red), for ribonucleotide base binding (‘nuc.’; gray), and of the phosphate cap (‘P-cap’; purple) are highlighted. Figure 2—source data 1.Data collection and refinement statistics. Figure 2—source data 2.Sequences used to calculate surface conservation and generate Weblogos.

To elucidate the molecular basis for these unique properties of Orn, we set out to gain a deeper understanding of the enzyme’s substrate specificity by determining Orn/substrate co-crystal structures. We initially determined the crystal structure of *P. aeruginosa* Orn (Orn*_Pa_*) with diribonucleotide substrate; however, crystal packing contacts prevented substrate binding (data not shown). Instead, we were able to crystallize two representative homologs, Orn*_Vc_* (Kamp et al., 2013) and the human REXO2 (also known as small fragment nuclease or Sfn [Bruni et al., 2013]), bound to the diribonucleotide pGpG (Figure 2A and B; Figure 2—source data 1). Both proteins purified free of divalent cation, which was sufficient to prevent catalysis in crystals. Superimposing the two structures indicates their identical fold (rmsd of 0.75 Å for the protomer) (Figure 2—figure supplement 1A), which is preserved in the substrate-free Orn structure determined previously (rmsd of 0.54 Å for the protomer) (Figure 2—figure supplement 1A) (Chin et al., 2006). The pGpG-bound structures reveal a narrow active site that is lined by the conserved acidic residues of the signature DEDD motif (D^12^, E^14^, and D^112^ of Orn*_Vc_*; D^15^, E^17^, and D^115^ of REXO2) and the general base H^158^ or H^162^ in Orn*_Vc_* or REXO2, respectively (Figure 2A and B and Figure 2—figure supplement 1B and Figure 2—source data 2). In Orn*_Vc_*, the bases of the diribonucleotide buttress against aromatic residues W^61^ and Y^129^, the latter being contributed from the second half-side of the dimeric enzyme. The corresponding residues, W^64^ and Y^132^, are conserved in REXO2. Residue L^18^ in Orn*_Vc_* (L^21^ in REXO2) wedges in-between the two bases. Most notably, residues S^108^, R^130^, S^135^ and the hydroxyl group on Y^129^ (S^111^, R^133^, S^138^, and Y^132^ in REXO2) form hydrogen bonds with the 5' phosphate of pGpG, capping the substrate. This phosphate cap creates a major constriction of the active site, which is not observed in structurally related 3'−5' exoribonucleases such as RNase T or ExoI (Figure 2C and Figure 2—figure supplement 1 and Figure 2—source data 2) (Hsiao et al., 2011; Korada et al., 2013; Hsiao et al., 2012). RNase T and ExoI accommodate longer RNA substrates, facilitated by an expansive active site. The structural analysis correlates closely with sequence conservation of the phosphate cap motif, which is strict in Orn homologs but divergent in RNase T proteins (Figure 2D and Figure 2—source data 2). Our structural analysis also suggests that modifications at the bases may be tolerated consistent with the observation that all native bases can be accommodated at the active site (Figure 2—figure supplement 2). Modifications at the sugar or termini beyond a terminal 5’-phosphate will likely fit sub-optimally if at all.

**Figure 2—figure supplement 1.**
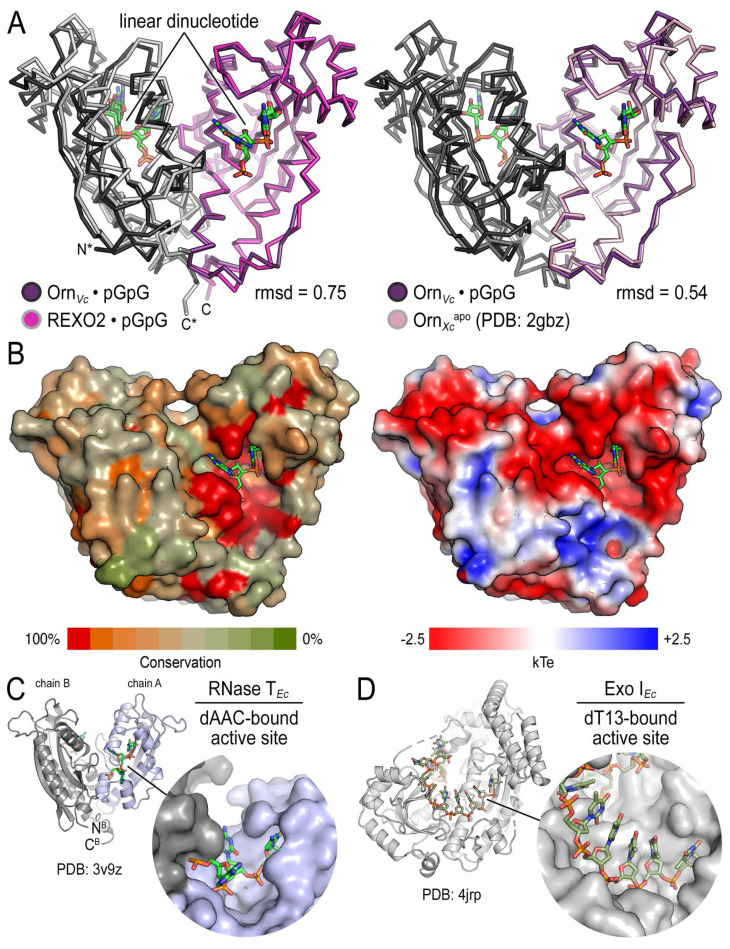
Structural comparison of Orn orthologs, RNase T and Exo I. (**A**) Superposition of Orn*_Vc_* with REXO2 (left) and the substrate-free state of Orn from *Xanthomonas campestris* (PDB 2gbz; Chin et al., 2006) (right). Rmsd values are reported for the alignment of a monomer. (**B**) Surface properties of Orn enzymes. An alignment of Orn orthologs was used to map conservation scores onto the solvent accessible surface of Orn*_Vc_* (left). High degree of conservation overlaps with the acidic active site observed in the electrostatic potential map calculated with the APBS software (Baker et al., 2001) and based on Orn*_Vc_* (right). (**C**) Active-site view of *E. coli* RNase T bound to dAAC (PDB 3v9z; Hsiao et al., 2012). (**D**) Active-site view of *E. coli* Exo I bound to dT13 (PDB 4jrp; Korada et al., 2013).

**Figure 2—figure supplement 2.**
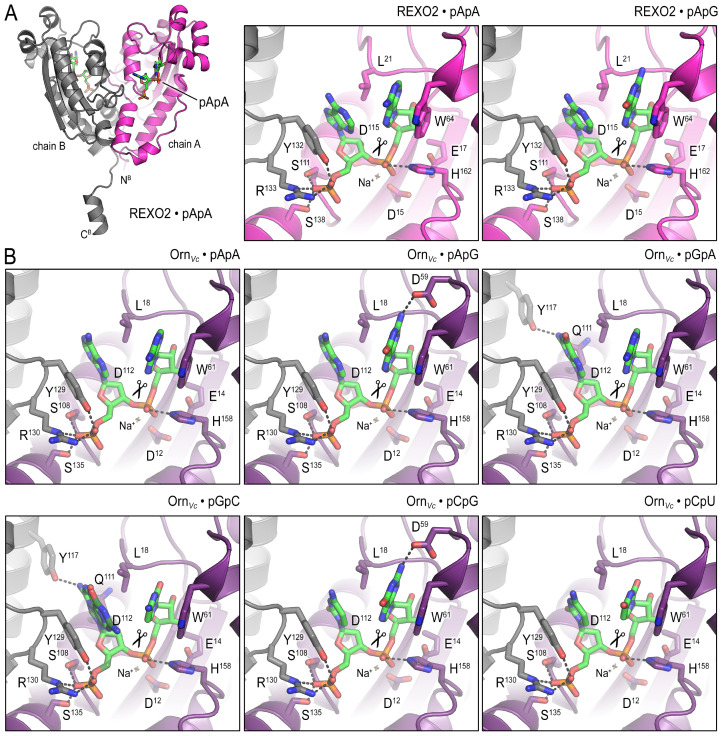
Structural comparison of Orn*_Vc_* and REXO2 bound to diverse diribonucleotides. (**A**) Crystal structures of REXO2 bound to pApA and pApG. The structural overview (left) shows a C-terminal helix that is unique to Orn orthologs in higher eukaryotes and in the current crystal forms is stabilized by crystal packing contacts. (**B**) Crystal structures of Orn*_Vc_* bound to di-purine (pApA, pApG, pGpA), purine-pyrimidine (pGpC, pCpG), and di-pyrimidine (pCpU) substrates. Views are identical to those shown in Figure 2 for the REXO2:pGpG and Orn:pGpG complexes.

Any attempts to obtain structures of Orn*_Vc_* with longer RNA substrates (3–5 bases in length) only resolved diribonucleotides at the active site (data not shown), further suggesting a narrow active site that is selective for dinucleotide species. These results are consistent with two recent reports of structural data for *Colwellia psychrerythraea* Orn and REXO2 bound to substrates (Lee et al., 2019; Chu et al., 2019). In the case of Orn*_Cp_*, crystallization was attempted with a 5-mer RNA; however, only two bases were resolved at the active site (Lee et al., 2019). A similar observation was made with REXO2 for RNA substrates, but a complex with a longer DNA-based ligand was obtained (Chu et al., 2019), which may not allow conclusions for RNA substrates. In the same report, RNase activity against longer RNA was observed with REXO2, but required a 20x molar excess of enzyme over RNA, likely not representing physiological conditions (Chu et al., 2019).

### The phosphate cap is required for diribonuclease activity

**Figure 3.**
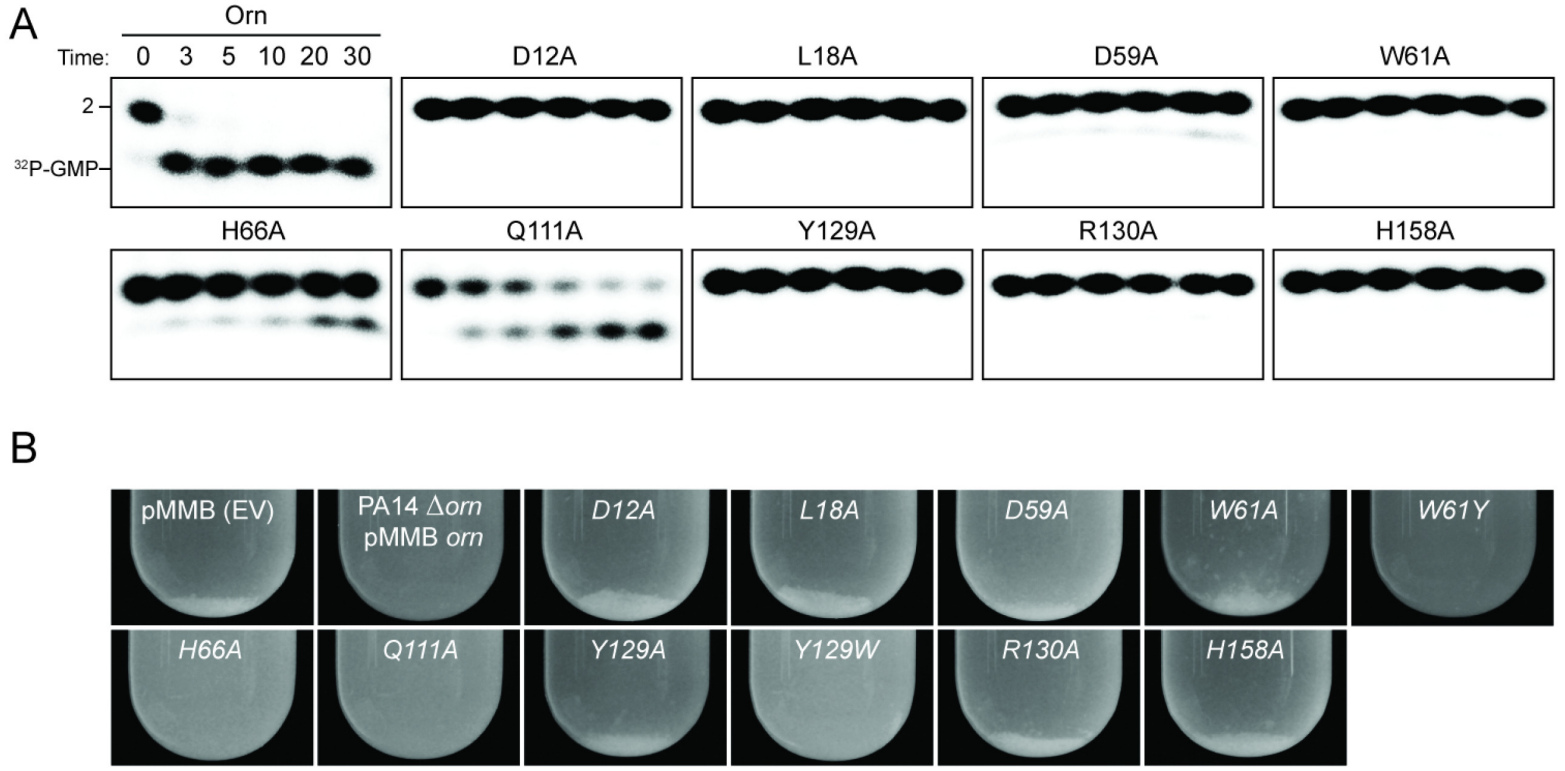
Functional importance of active-site and phosphate-cap residues for Orn function. (**A**) In vitro enzyme activity. Degradation of ^32^P-pGpG (1 μM) by purified wild-type Orn*_Vc_* or variants with alanine substitutions (5 nM) at the indicated sites was assessed. Samples were stopped at the indicated times (min) and analyzed by denaturing 20% PAGE. Representative gel images are shown with the indicated RNA size. Figure 3—figure supplement 4 shows the graphs of the means and SD of three independent experiments. (**B**) In vivo activity of alanine substituted *orn_Vc_* alleles to complement *P. aeruginosa Δorn*. Overnight cultures of the indicated strains were allowed to stand for 10 min without agitation to allow bacterial aggregates to sediment. Representative images of the cultures of triplicated assays are shown.

To assess the impact on catalysis of the phosphate cap in comparison to other active site residues identified in the structural analysis, we introduced specific single-point mutations into Orn*_Vc_* (Figure 3). All protein variants expressed stably in *P. aeruginosa* (Figure 3—figure supplement 2). For two representative point mutants with alanine substitutions at a phosphate-cap or active-site/DEDD-motif residue, Orn*_Vc_*-R^130^A or Orn*_Vc_*-D^12^A, respectively, we showed that the purified proteins form stable dimers in solution, comparable to wild-type Orn*_Vc_* (Figure 3—figure supplement 3). Purified protein variants were evaluated for pGpG degradation. Mutations of the central phosphate cap residue R^130^ to alanine led to complete loss of catalytic activity, comparable to mutants in the DEDD active site motif (Figure 3A and Figure 3—figure supplement 4) (Orr et al., 2015). These protein variants were similarly unable to cleave the 7-mer (Figure 1—figure supplement 1). We next asked whether Orn*_Vc_* with specific point mutations could complement the deletion of *orn* in *P. aeruginosa. P. aeruginosa* ∆*orn* accumulates pGpG that in turn inhibits c-di-GMP-specific phosphodiesterases (Cohen et al., 2015; Orr et al., 2015). As a net result, c-di-GMP accumulates in these cells, an effect that is associated with a hyper-biofilm and cell aggregation phenotype. While expression of wild-type Orn*_Vc_* complements the *P. aeruginosa* ∆*orn* resulting in a dispersed culture, complementation with variants that carry mutations in either the active site or phosphate cap residues results in cell aggregation indistinguishable from the ∆*orn* phenotype (Figure 3B). Together, these experiments demonstrate that an intact phosphate cap is required for enzyme function.

**Figure 3—figure supplement 1.**
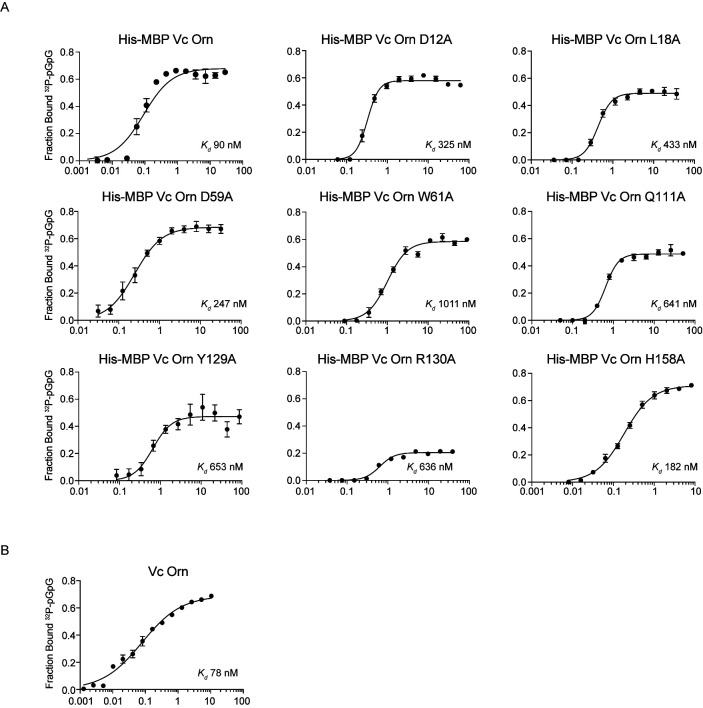
Contribution of catalytic residues and His_10_-MBP tag to binding interaction between Orn*_Vc_* and pGpG. The dissociation constants (*K_d_*) of indicated purified (**A**) His_10_-MBP-Orn*_Vc_* variants and (**B**) untagged Orn*_Vc_* to pGpG were measured by DRaCALA. Fraction bound from data was plotted and determined by nonlinear regression. All data shown represent average and SD of three independent experiments.

**Figure 3—figure supplement 2.**
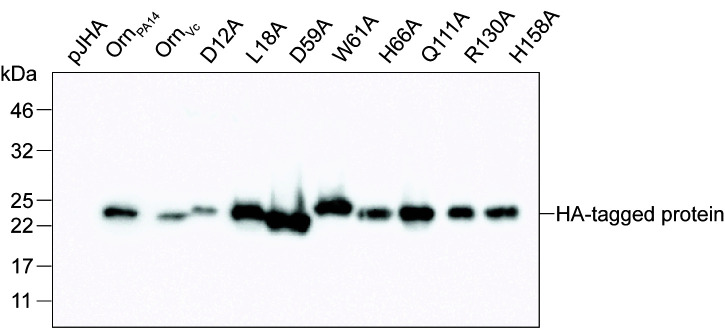
Immunoblot analysis of Orn in *P. aeruginosa* ∆*orn* expressing indicated *orn* alleles. Proteins in whole cell lysates of strains expressing the indicated HA-tagged *orn* allele were separated by 12% SDS-PAGE and probed with anti-HA antibodies. All images were performed in duplicate independently. A representative image of two independent experiments is shown.

**Figure 3—figure supplement 3.**
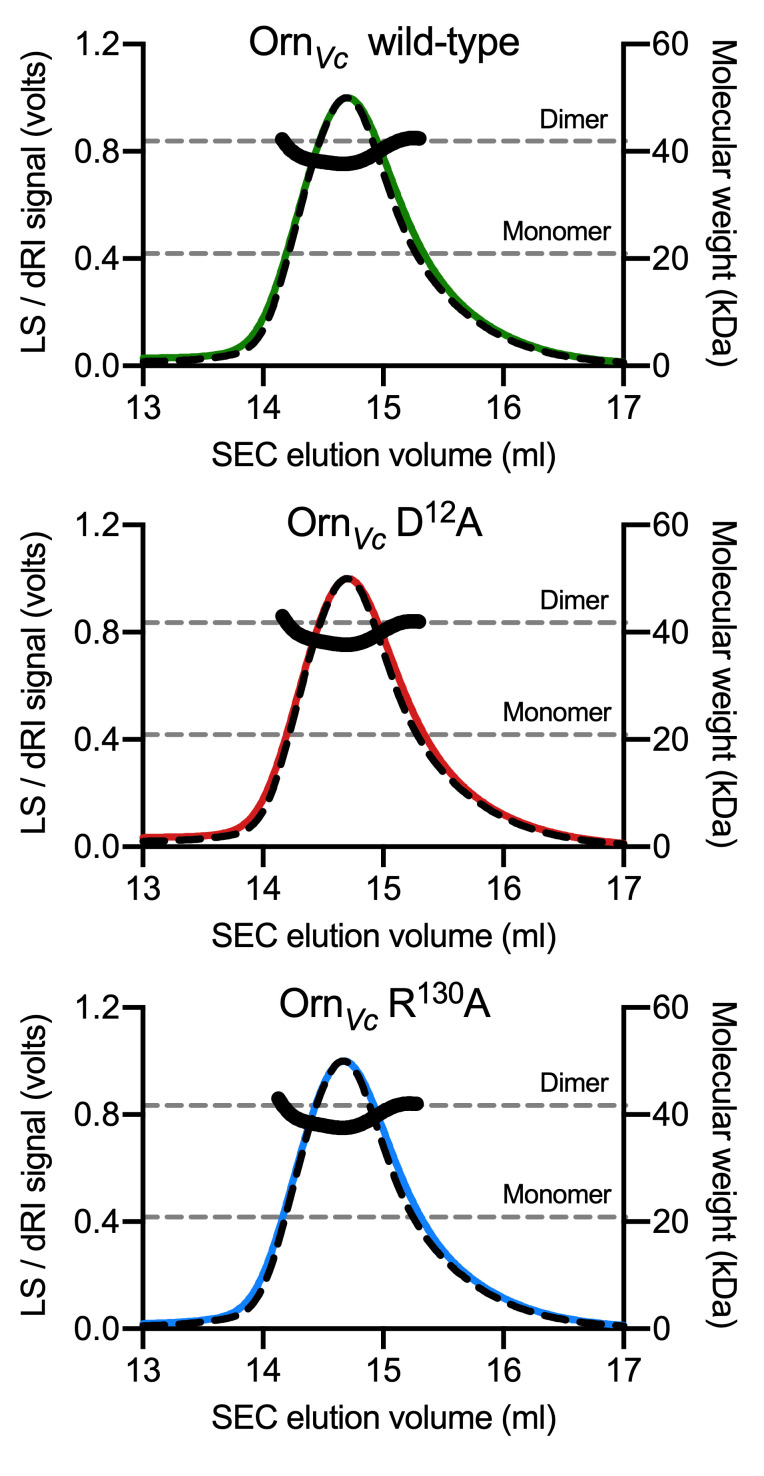
Molecular weight determination indicates that Orn*_Vc_* variants with point mutations at the phosphate cap and active site remain dimeric. (**A**) Absolute molecular weights (black data points across elution peaks are plotted on the right axis; theoretical monomer and dimer molecular weights, horizontal dashed lines) of wild-type Orn*_Vc_* and point mutants were determined using SEC-MALS (90°-light scattering: colored, solid lines; refractive index signal: black, dashed lines; plotted on the left axis).

**Figure 3—figure supplement 4.**
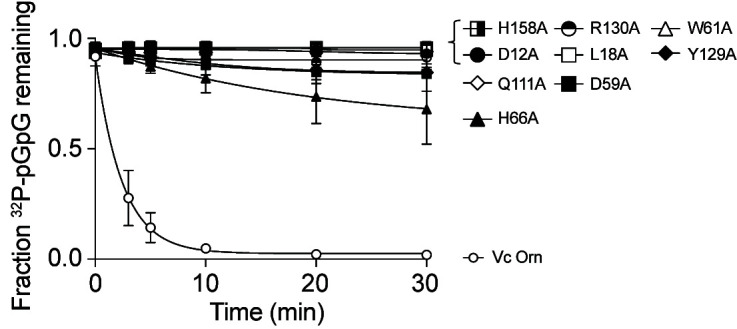
pGpG degradation by purified wild-type Orn*_Vc_* or variants with indicated alanine substitutions. 1 µM pGpG was treated with 5 nM of the indicated Orn protein and sampled at indicated time points. The graphs show quantification of three independent data sets, of which representative radiographs are shown in Figure 3A. Figure 3—figure supplement 4—source data 1.Numerical data for Figure 3—figure supplement 4.

### Interaction of Orn with substrates

**Figure 4.**
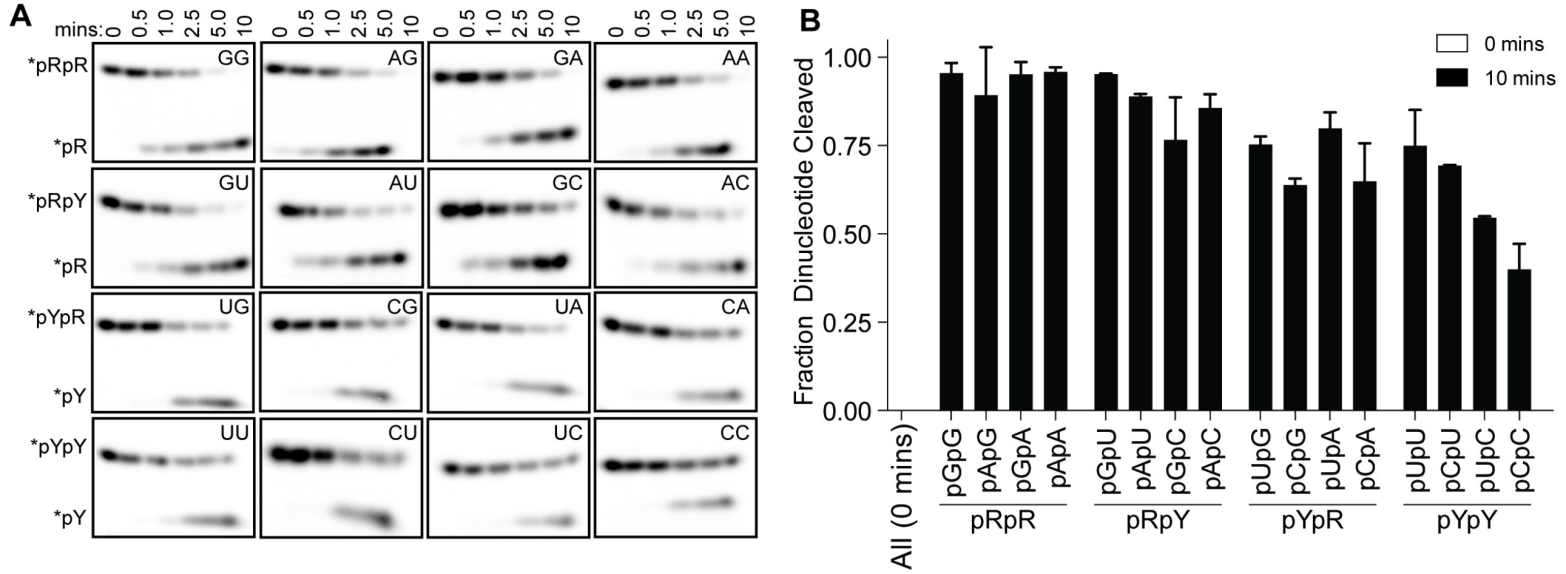
Orn*_Vc_* cleaves all diribonucleotides. (**A**) Orn*_Vc_* (5 nM) was incubated with di-purine (pRpR), purine-pyrimidine (pRpY or pYpR), or di-pyrimidine (pYpY) diribonucleotides (1 μM) containing the corresponding ^32^P-labeled RNA tracer. Aliquots of each reaction were stopped at the indicated times (min) and assessed by denaturing 20% PAGE. (**B**) Quantification of the intensities of bands corresponding to the amount of diribonucleotide cleaved at the 10 min time point. Results are the average and SD of duplicate independent experiments. Orn cleaves all diribonucleotides to nucleoside monosphosphates, albeit to varying extents. Diribonucleotides consisting of two purines (pRpR) were hydrolyzed most efficiently, with over 90% of starting RNAs processed by 10 min. A majority (>75%) of diribonucleotides with a 5' purine (pRpY) were also processed by the 10 min endpoint. However, diribonucleotides with a 5' pyrimidines exhibited moderately reduced levels of cleavage. Di-pyrimidine (pYpY) substrates, and in particular pUpC and pCpC, showed the slowest turnover from the substrates tested. These results demonstrate that while all diribonucleotides are acceptable substrates for Orn, the enzyme is likely to exhibit moderate preferences for diribonucleotides that contain a 5' purine. Figure 4—source data 1.Numerical data for Figure 4B.

Structures of Orn*_Vc_* (and REXO2) with different diribonucleotides, including di-purine, di-pyrimidine and mixed substrates, revealed identical binding poses (Figure 2—figure supplement 2). Additional hydrogen bonding between purine residues and Orn in the 3' or 5' position correlates with a small, but detectable preference of Orn*_Vc_* for purine-containing substrates (Figure 4). Together, the structural analysis uncovered Orn’s mode of substrate binding, which is conserved from bacteria to humans and indicates a unique selection for linear diribonucleotides with a 5’ phosphate. The requirement for the 5’ phosphate was tested with GpG as a competitor for Orn binding and cleavage of pGpG. Even in excess GpG, Orn bound pGpG and cleaved pGpG similar to untreated control (Figure 4—figure supplement 1A B) supporting the importance of the 5’ phosphate cap.

**Figure 4—figure supplement 1.**
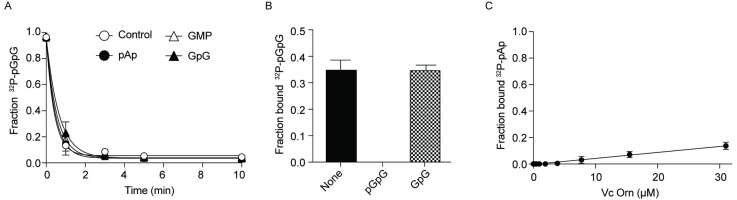
Effect of GpG and pAp on Orn activity. (**A**) Cleavage of 130 nM ^32^P-pGpG by 5 nM Orn in the absence or presence of unlabeled pAp, GpG or GMP (100 µM). (**B**) Fraction bound of ^32^P-pGpG to 200 nM Orn in presence of no competitor, 100 µM pGpG or 100 µM GpG. (**C**) Fraction bound of ^32^P-pAp to different concentration of Orn*_Vc_* was determined by DRaCALA. *K_d_* could not be measured as ^32^P-pAp binding to Orn was not saturated. (**B**) (**C**) All data shown represent duplicate-independent experiments. Figure 4—figure supplement 1—source data 1.Numerical data for Figure 4—figure supplement 1.

Another distinguishing factor between Orn and other exoribonucleases with similar activities is the apparent inhibition of Orn by 3′-phosphoadenosine 5′-phosphate (pAp), a metabolic byproduct of sulfate assimilation that accumulates upon lithium poisoning of pAp-phosphatase (Mechold et al., 2007). DHH/DHHA-type oligoribonucleases such as NrnA from *Bacillus subtilis*, *Mycobacterium tuberculosis* and *Mycoplasma pneumoniae* are capable of dephosphorylating the pAp mononucleotide and their activity against oligoribonucleotides is unaffected by pAp (Mechold et al., 2007; Postic et al., 2012). In contrast, pAp has been described as a competitive inhibitor for *E. coli* Orn and the human REXO2 (Mechold et al., 2006). However, the oligo-cytosine RNAs used in these prior studies were labeled at their 5’ end with a bulky fluorescent dye moiety (usually a cyanine or Cy-dye) (Cohen et al., 2015; Mechold et al., 2006); our aggregate data now indicate that these RNAs represent suboptimal substrates for Orn, given its stark requirement for a simple 5’ phosphate (Figure 2). This labeling strategy is therefore likely to incompletely assess native Orn activity by underestimating diribonuclease activity while also overestimating the effect of competitive inhibitors. Revisiting pAp binding to Orn, we show here that Orn*_Vc_* does not interact with radiolabeled pAp in the binding assay that was used to quantify oligoribonucleotide interactions with Orn (Figure 4—figure supplement 1C). Furthermore, unlabeled pAp failed to competitively inhibit degradation of radiolabeled pGpG to GMP (Figure 4—figure supplement 1A).

### Orn is the only diribonuclease in *P. aeruginosa* grown under laboratory conditions

**Figure 5.**
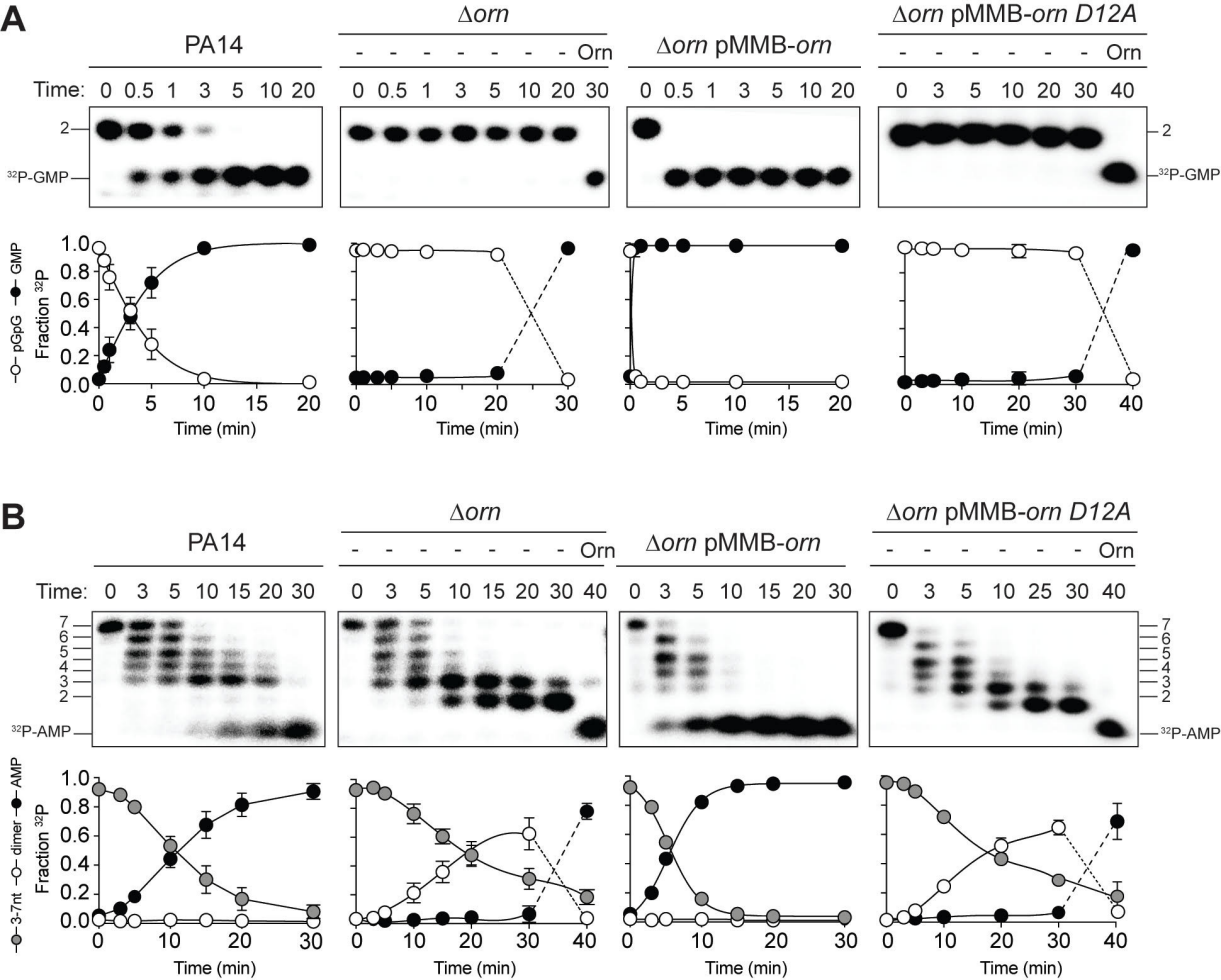
Orn acts as a diribonuclease in cell lysates. Degradation of ^32^P-GG (**A**) and ^32^P-AAAAAGG (**B**) by whole cell lysates of wild-type, *orn* mutant, *orn* mutant complemented with *orn_Vc_,* or *orn_Vc_ D^12^A*. For ∆*orn* and ∆*orn* complemented with *orn_Vc_ D^12^A*,100 nM of purified Orn*_Vc_* was added at 30 min time point and incubated for an additional 10 min. Samples were stopped at the indicated time and analyzed by 20% denaturing PAGE. Representative gel images of triplicated assays are shown with the indicated RNA size. Graphs show quantitation of triplicate data for indicated RNA species over time. Figure 5—source data 1.Numerical data for Figure 5.

All available literature assumes Orn is a 3’−5’ exoribonuclease that is responsible for processing of short (between 2 and 7) oligoribonucleotides. Yet our structural and biochemical data suggest that the enzyme exhibits such a striking preference for diribonucleotide substrates that the in vivo function of Orn as a general exoribonuclease should be reconsidered. Therefore, we developed experimental conditions to measure Orn’s activity in cellular extracts. The *orn* gene is required for viability in most **γ**-proteobacteria, including *V. cholerae*; however, it is not essential for growth of *P. aeruginosa* under most conditions (Cohen et al., 2015; Orr et al., 2015). Therefore, lysates were generated from *P. aeruginosa* strains, including parental PA14, ∆*orn*, and ∆*orn* complemented with *orn_Vc_*. 5'-^32^P-radiolabeled 2-mer or 7-mer RNA was then added to each of these lysates (Figure 5). Aliquots of the mixtures were removed and analyzed by urea denaturing 20% PAGE at varying time intervals. Extracts from parental PA14 digested the entire radiolabeled diribonucleotide in less than 5 min (Figure 5A). In contrast, lysates from strains lacking Orn failed to show any signs of diribonuclease activity even at longer time points. Similarly, complementation with catalytically inactive alleles of *orn* (D^12^A, L^18^A and H^158^A) also failed to clear the diribonucleotide (Figure 5 and Figure 5—figure supplement 1). Diribonuclease activity in the lysates could be restored by ectopic expression of *orn* from a self-replicating plasmid or by addition of purified Orn (Figure 5A). These data confirm that cellular Orn is required for degrading diribonucleotides and no other cellular RNase of *P. aeruginosa* can substitute for Orn activity under these laboratory growth conditions.

**Figure 5—figure supplement 1.**
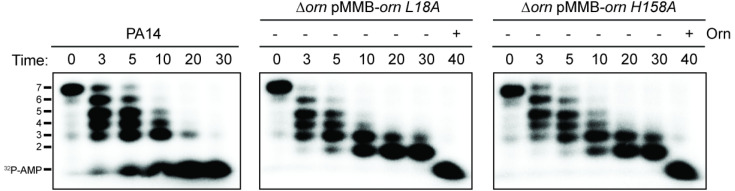
Catalytic residues of Orn*_Vc_* are required for degradation for ^32^P-AAAAAGG in whole-cell lysates. Degradation of ^32^P-AAAAAGG by whole-cell lysates of the indicated strain (PA14, ∆*orn* mutant complemented with pMMB-*orn_Vc_ L^18^A* or pMMB-*orn_Vc_ H^158^A*. For ∆*orn* expressing *orn_Vc_ L^18^A* or *orn_Vc_ H^158^A*, 100 nM of purified, wild-type Orn*_Vc_* protein was added at the 30 min time point and reactions were stopped 10 min thereafter. Samples were stopped at indicated time and analyzed by denaturing 20% PAGE. Representative gel images of two independent experiments are shown.

When the ^32^P-7-mer RNA substrate was incubated in extracts from parental PA14 it was digested to a ladder of degradative intermediates including 6-mer, 5-mer, 4-mer and 3-mer RNAs (Figure 5B). Of note, while these intermediates and the final mononucleotide product accumulated over time, the 2-mer intermediate was never observed over the time course. In contrast, lysates from the ∆*orn* mutant specifically accrued the diribonucleotide intermediate with no apparent production of its mononucleotide products. Ectopic expression of plasmid-borne *orn*, but not *orn D^12^A*, restored the degradation of the 7-mer to mononucleotides and the diribonucleotide intermediate could no longer be observed. Furthermore, the diribonucleotide intermediate that accumulated in the ∆*orn* lysate was fully processed upon addition of purified Orn protein (Figure 5B). Together, these results show that *P. aeruginosa* accumulates a bottleneck of diribonucleotide intermediates in a ∆*orn* background, which is only resolved upon addition of Orn. Degradation of RNA fragments with three or more residues by Orn is negligible in a cellular context, considering that ∆*orn* lysates preserve nuclease activities for the processing of RNAs down to diribonucleotides. From these aggregate data, we propose that Orn functions not as an oligoribonuclease as stated in the literature but instead functions as a specialized ribonuclease of diribonucleotide substrates (i.e. a ‘diribonuclease’).

### The diribonucleotide pool affects *P. aeruginosa* growth in addition to c-di-GMP signaling

**Figure 6.**
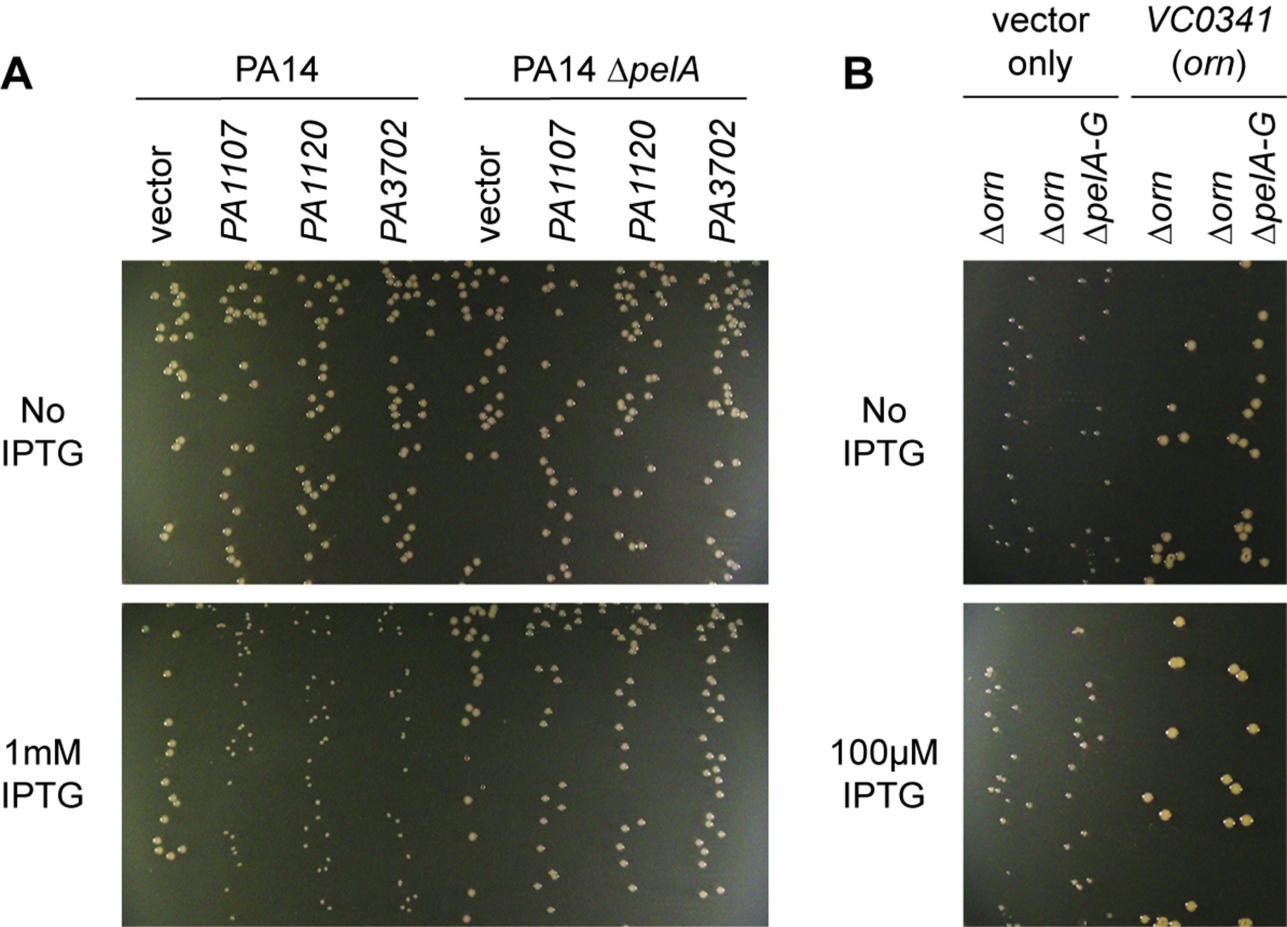
Small-colony phenotype of ∆*orn* is independent of c-di-GMP signaling. Bacterial cultures were diluted and dripped on LB agar plates with the indicated concentration of IPTG. After overnight incubation, representative images of the plates were taken and shown for (**A**) PA14 and PA14 ∆*pelA* harboring pMMB vector with the indicated diguanylate cyclase gene and (**B**) PA14 ∆*orn* and PA14 ∆*orn* ∆*pelA-G* with pMMB and pMMB-*orn*. Experiments were performed in triplicate.

We noticed that PA14 ∆*orn* had reduced growth on agar plates that is reminiscent of a small colony variant (SCV) phenotype (Figure 6) (D'Argenio et al., 2002). The SCV phenotype has been attributed previously to increased c-di-GMP levels (Hickman et al., 2005). C-di-GMP binds to FleQ and activates transcription of the *pel* operon (Baraquet et al., 2012; Matsuyama et al., 2016). In addition, c-di-GMP binds to the c-di-GMP-receptor PelD to increase the biosynthesis of the pel exopolysaccharide (Lee et al., 2007), which enhances cell aggregation leading to a compact SCV morphology. Previous reports had shown that *P. aeruginosa* ∆*orn* is unable to clear pGpG, which results in the elevation of c-di-GMP signaling and pel-dependent cell aggregation and biofilm mass (Cohen et al., 2015; Orr et al., 2015).

**Figure 6—figure supplement 1.**
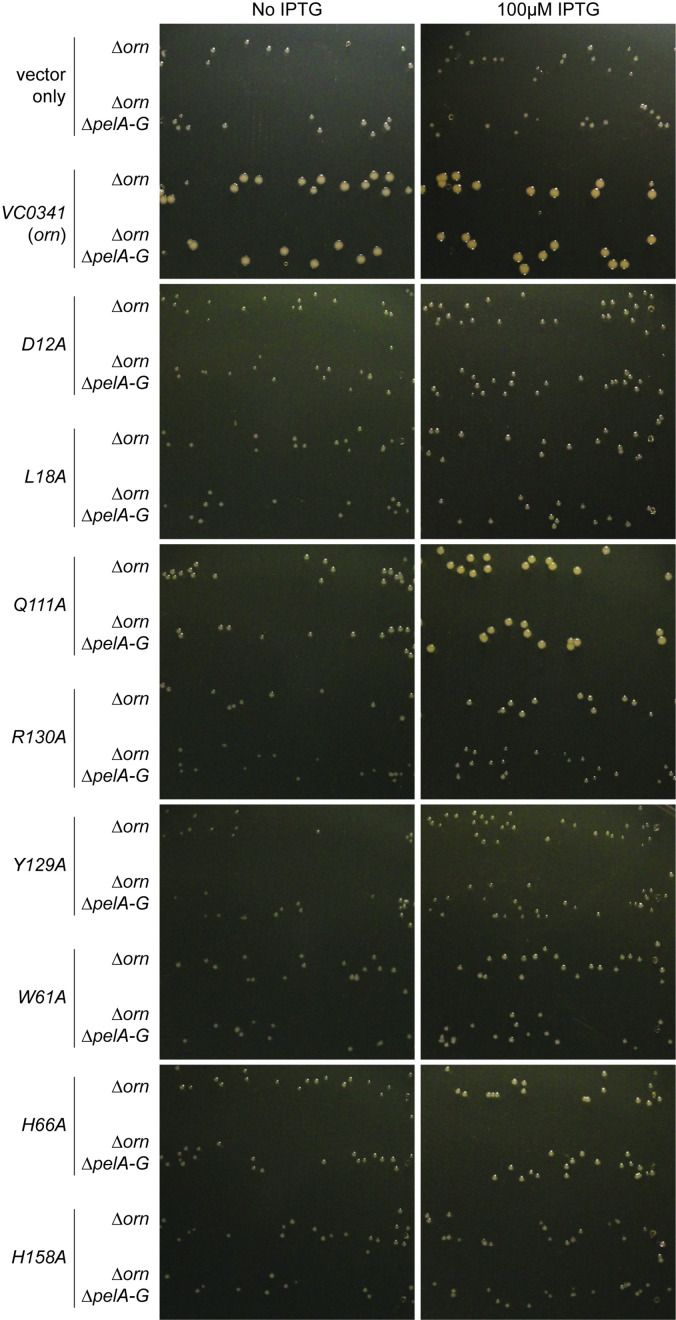
Small-colony phenotype of ∆*orn* is complemented by functional *orn_Vc_* alleles. Bacterial cultures were diluted, 10 µL was spotted on LB agar plates with the indicated concentration of IPTG and drip to form parallel lines of bacteria. After overnight incubation, representative images of the plates were taken of PA14 ∆*orn* and PA14 ∆*orn* ∆*pelA-G* with pMMB alone or containing the indicated allele of *orn_Vc_*. Experiments were performed in triplicate and one representative image of the plates is shown.

**Figure 6—figure supplement 2.**
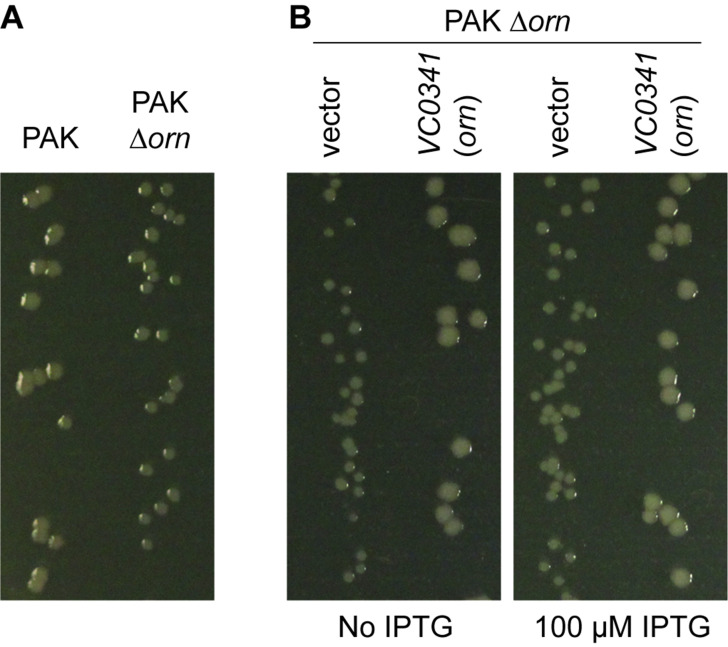
*P. aeruginosa* PAK *∆orn* mutants show a small-colony phenotype. Indicated bacterial strains were diluted and dripped on (**A**) LB agar plates or (**B**) LB agar plates containing 50 µg/mL carbenicillin with indicated concentration of IPTG and incubated overnight at 37°C. All experiments were performed in triplicate and one representative image of the plates is shown.

To determine whether the functional impact of diribonucleotide build-up is due specifically to an increased pGpG level and its effect on c-di-GMP, we asked whether the SCV formation in the ∆*orn* mutant is dependent on c-di-GMP processes. First, we confirmed that SCV formation in PA14 can be induced by elevated c-di-GMP levels through overexpression of diguanylate cyclases, such as PA1107 (*roeA*), PA1120 (*yfiN*/*tpbB*) and PA3702 (*wspR*) (Figure 6A) (Hickman et al., 2005; Kulasakara et al., 2006; Ueda and Wood, 2009; Malone et al., 2010). As expected, the effect of c-di-GMP is due to increased production of the pel exopolysaccharide and biofilm formation since the PA14 ∆*pelA* mutant maintained normal colony morphology even when these diguanylate cyclases were overexpressed (Figure 6A). To determine whether the increase in the pool of pGpG and c-di-GMP is the only reason for small-colony growth in the *∆orn* mutant, a ∆*orn* ∆*pel* double mutant was tested. When ∆*orn* ∆*pel* was grown on agar, it also had an apparent SCV phenotype (Figure 6B). Complementation of ∆*orn* and ∆*orn* ∆*pel* with active alleles of *orn* restored normal colony morphology, whereas inactive *orn* alleles failed to complement (Figure 6—figure supplement 1). In every case, the colony morphology was the same between ∆*orn* and ∆*orn* ∆*pel* indicating a second pathway that can restrict colony growth, but in this case independent of *pel* and c-di-GMP. Similar results were obtained for *P. aeruginosa* PAK strain (Figure 6—figure supplement 2). These results indicate that increased pools of one or more of the diribonucleotides function to cause small-colony growth in ∆*orn* in addition to the actions of pGpG on c-di-GMP signaling.

### Conclusion

**Figure 7.**
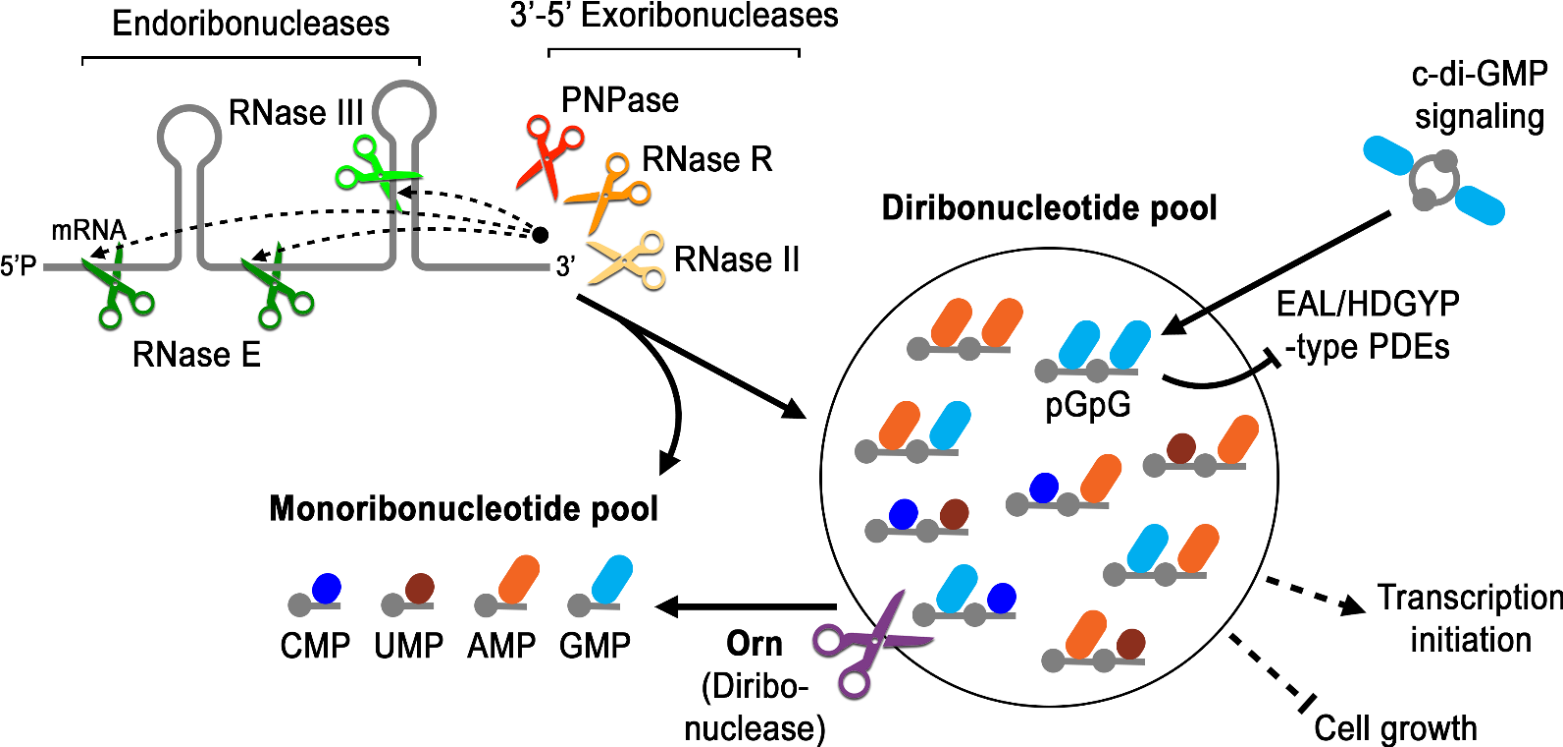
Model for Orn’s cellular function as a diribonuclease. RNA degradation is initiated by fragmentation via endoribonucleases (e.g. RNase E and RNase III) that cleave unstructured or structured RNA sequences. RNA fragments are processed further at their 3’ termini by 3’−5’ exoribonucleases (e.g. PNPase, RNase R, and RNase II). Their combined activity produces mononucleotides and various terminal diribonucleotides from the original RNA fragments. The pGpG (GG) linear diribonucleotide is also produced by linearization of c-di-GMP by specific phosphodiesterases, EAL- and HD-GYP-domain-containing enzymes, which terminate c-di-GMP signaling. In *Pseudomonas aeruginosa* and likely other organisms that rely on Orn for growth, Orn is the only diribonuclease that cleaves diribonucleotides to mononucleotides. In the absence of Orn, diribonucleotides accumulate with a drastic impact on cellular physiology, ranging from transcriptional changes, small-colony phenotypes and growth arrest, depending on the organism. Orn is also unique because it acts as the second phosphodiesterase in the degradation of c-di-GMP by clearing the pGpG intermediate. In an *orn* mutant, c-di-GMP levels are elevated through feedback inhibition of the c-di-GMP-degrading phosphodiesterases by pGpG, leading to the associated biofilm phenotypes.

Orn is unique amongst exoribonucleases because it is essential in some γ-proteobacteria and is required to degrade the pGpG intermediate in c-di-GMP signaling. Yet the molecular and structural requirements for Orn were unknown. Our studies reveal that Orn is a dedicated diribonuclease in cells. This appears to be driven by a catalytic site that is restricted by a cap that mediates multiple interactions with the 5’ phosphate of diribonucleotide substrates. This constriction prevents longer substrates from binding with high affinity, rendering them poor substrates for catalytic cleavage. The discovery that Orn acts as a dedicated diribonuclease indicates that this activity clears a specific diribonucleotide bottleneck in global RNA degradation (Figure 7). Prior studies have shown that *orn* depletion can lead to accumulation of diribonucleotides and longer oligoribonucleotides in some cellular backgrounds (Ghosh and Deutscher, 1999). The increase in oligoribonucleotides longer than dimers in cells may occur through feedback inhibition of other enzymes in the RNA degradation pathway, in analogy to the impact of pGpG on c-di-GMP-degrading phosphodiesterases and c-di-GMP signaling (Figure 7) (Cohen et al., 2015; Orr et al., 2015). Whether the effect of diribonucleotides on essentiality is due to general processes such as RNA degradation, altered transcription (Goldman et al., 2011; Druzhinin et al., 2015; Vvedenskaya et al., 2012), specific interactions with essential proteins, or a combination of these remains to be evaluated in this context. Our studies therefore reveal a key step in RNA degradation, the enzymatic cleavage of diribonucleotides into mononucleotides, and set the stage to address how diribonucleotide accumulation is detrimental to cell survival.

## Materials and methods

**Key resources table:** 

Reagent type or resource	Designation	Source or reference	Identifiers	Additional information
*Strain (P. aeruginosa)*	PA14	Rahme et al., 1995; PMID 7604262		
*Strain (P. aeruginosa)*	*PA14 ∆pelA*	Lee et al., 2007; PMID: 17824927		
*Strain (P. aeruginosa)*	*PA14 ∆orn*	Orr et al., 2015; PMID: 26305945		
*Strain (P. aeruginosa)*	*PA14 ∆orn ∆pelA-G*	Orr et al., 2015; PMID: 26305945		
*Strain (P. aeruginosa)*	PAK	Bradley, 1974; PMID: 4206974		
*Strain (P. aeruginosa)*	*PAK ∆orn*	This study		Generated using pEX-Gn-∆orn (PAK)
Strain (*E. coli*)	XL10-Gold	Agilent		
Strain (*E. coli*)	Stellar cells	Takara/Clontech		
Strain (*E. coli*)	BL21(DE3)	New England Biolabs		
Strain (*E. coli*)	NEB T7Iq	New England Biolabs		
Genetic reagent (plasmid)	pET28-His6-SUMO-OrnVc	This study		cloned from custom DNA fragment (see below) for purification of His6-SUMO-OrnVc, OrnVc
Genetic reagent (plasmid)	pET28-His6-SUMO-Rexo2	This study		cloned from custom DNA fragment (see below) for purification of His6-SUMO-Rexo2, Rexo2
Genetic reagent (plasmid)	pDONR221-VC0341 (ornVc)	Rolfs et al., 2008; PMID: 18337508		
Genetic reagent (plasmid)	pDONR221-ornVc D12A	This study		site-directed mutagenesis to introduce alanine at D12 position
Genetic reagent (plasmid)	pDONR221-ornVc L18A	This study		site-directed mutagenesis to introduce alanine at L18 position
Genetic reagent (plasmid)	pDONR221-ornVc D59A	This study		site-directed mutagenesis to introduce alanine at D59 position
Genetic reagent (plasmid)	pDONR221-ornVc Q111A	This study		site-directed mutagenesis to introduce alanine at Q111 position
Genetic reagent (plasmid)	pDONR221-ornVc R130A	This study		site-directed mutagenesis to introduce alanine at R130 position
Genetic reagent (plasmid)	pDONR221-ornVc Y129A	This study		site-directed mutagenesis to introduce alanine at Y129 position
Genetic reagent (plasmid)	pDONR221-ornVc Y129W	This study		site-directed mutagenesis to introduce tryptophan at Y129 position
Genetic reagent (plasmid)	pDONR221-ornVc W61A	This study		site-directed mutagenesis to introduce alanine at W61 position
Genetic reagent (plasmid)	pDONR221-ornVc W61Y	This study		site-directed mutagenesis to introduce tyrosine at W61 position
Genetic reagent (plasmid)	pDONR221-ornVc H66A	This study		site-directed mutagenesis to introduce alanine at H66 position
Genetic reagent (plasmid)	pDONR221-ornVc H158A	This study		site-directed mutagenesis to introduce alanine at H158 position
Genetic reagent (plasmid)	pVL847-ornVc	Orr et al., 2015; PMID: 26305945		OrnVc from pDONR cloned into pVL847 for purification of His-MBP-OrnVc, OnrVc
Genetic reagent (plasmid)	pVL847-ornVc D12A	This study		D12A from pDONR cloned into pVL847 for purification of His-MBP-OrnVc D12A, OrnVc D12A
Genetic reagent (plasmid)	pVL847-ornVc L18A	This study		L18A from pDONR cloned into pVL847 for purification of His-MBP-OrnVc L18A, OrnVc L18A
Genetic reagent (plasmid)	pVL847-ornVc D59A	This study		L18A from pDONR cloned into pVL847 for purification of His-MBP-OrnVc D59A, OrnVc D59A
Genetic reagent (plasmid)	pVL847-ornVc Q111A	This study		Q111A from pDONR cloned into pVL847 for purification of His-MBP-OrnVc Q111A, OrnVc Q111A
Genetic reagent (plasmid)	pVL847-ornVc R130A	This study		R130A from pDONR cloned into pVL847 for purification of His-MBP-OrnVc R130A, OrnVc R130A
Genetic reagent (plasmid)	pVL847-ornVc Y129A	This study		Y129A from pDONR cloned into pVL847 for purification of His-MBP-OrnVc Y129A, OrnVc Y129A
Genetic reagent (plasmid)	pVL847-ornVc W61A	This study		W61A from pDONR cloned into pVL847 for purification of His-MBP-OrnVc W61A, OrnVc W61A
Genetic reagent (plasmid)	pVL847-ornVc H66A	This study		H66A from pDONR cloned into pVL847 for purification of His-MBP-OrnVc H66A, OrnVc H66A
Genetic reagent (plasmid)	pVL847-ornVc H158A	This study		H158A from pDONR cloned into pVL847 for purification of His-MBP-OrnVc H158A, OrnVc H158A
Genetic reagent (plasmid)	pMMB-Gn	Fürste et al., 1986; PMID: 3549457		
Genetic reagent (plasmid)	pMMB-Gn-PA1107	Kulasakara et al., 2006; PMID: 16477007		
Genetic reagent (plasmid)	pMMB-Gn-PA1120	Kulasakara et al., 2006; PMID: 16477007		
Genetic reagent (plasmid)	pMMB-Gn-PA3702 (wspR)	Kulasakara et al., 2006; PMID: 16477007		
Genetic reagent (plasmid)	pMMB-Ap	Fürste et al., 1986; PMID: 3549457		
Genetic reagent (plasmid)	pMMB-Ap-VC0341 (ornVc)	This study		Orn from pDONR cloned into pMMB for expressing in *P. aeruginosa*
Genetic reagent (plasmid)	pMMB-Ap-ornVc D12A	This study		D12A from pDONR cloned into pMMB for expressing in *P. aeruginosa*
Genetic reagent (plasmid)	pMMB-Ap-ornVc L18A	This study		L18A from pDONR cloned into pMMB for expressing in *P. aeruginosa*
Genetic reagent (plasmid)	pMMB-Ap-ornVc Q111A	This study		Q111A from pDONR cloned into pMMB for expressing in *P. aeruginosa*
Genetic reagent (plasmid)	pMMB-Ap-ornVc R130A	This study		R130A from pDONR cloned into pMMB for expressing in *P. aeruginosa*
Genetic reagent (plasmid)	pMMB-Ap-ornVc Y129A	This study		Y129A from pDONR cloned into pMMB for expressing in *P. aeruginosa*
Genetic reagent (plasmid)	pMMB-Ap-ornVc Y129W	This study		Y129W from pDONR cloned into pMMB for expressing in *P. aeruginosa*
Genetic reagent (plasmid)	pMMB-Ap-ornVc W61A	This study		W61A from pDONR cloned into pMMB for expressing in *P. aeruginosa*
Genetic reagent (plasmid)	pMMB-Ap-ornVc W61Y	This study		W61Y from pDONR cloned into pMMB for expressing in *P. aeruginosa*
Genetic reagent (plasmid)	pMMB-Ap-ornVc H66A	This study		H66A from pDONR cloned into pMMB for expressing in *P. aeruginosa*
Genetic reagent (plasmid)	pMMB-Ap-ornVc H158A	This study		H158A from pDONR cloned into pMMB for expressing in *P. aeruginosa*
Genetic reagent (plasmid)	pEX-Gn-∆orn (PAK)	This study		
Genetic reagent (plasmid)	pJHA-Gn	This study		
Genetic reagent (plasmid)	pJHA-Gn-ornPa	This study		OrnPA (Orr, et al, PNAS 2015) cloned into pJHA for expressing in *P. aeruginosa*
Genetic reagent (plasmid)	pJHA-Gn-ornVc	This study		Orn from pDONR cloned into pJHA for expressing in *P. aeruginosa*
Genetic reagent (plasmid)	pJHA-Gn-ornVc D12A	This study		D12A from pDONR cloned into pJHA for expressing in *P. aeruginosa*
Genetic reagent (plasmid)	pJHA-Gn-ornVc L18A	This study		L18A from pDONR cloned into pJHA for expressing in *P. aeruginosa*
Genetic reagent (plasmid)	pJHA-Gn-ornVc D59A	This study		Q111A from pDONR cloned into pJHA for expressing in *P. aeruginosa*
Genetic reagent (plasmid)	pJHA-Gn-ornVc W61A	This study		R130A from pDONR cloned into pJHA for expressing in *P. aeruginosa*
Genetic reagent (plasmid)	pJHA-Gn-ornVc H66A	This study		Y129A from pDONR cloned into pJHA for expressing in *P. aeruginosa*
Genetic reagent (plasmid)	pJHA-Gn-ornVc Q111A	This study		W61A from pDONR cloned into pJHA for expressing in *P. aeruginosa*
Genetic reagent (plasmid)	pJHA-Gn-ornVc R130A	This study		H66A from pDONR cloned into pJHA for expressing in *P. aeruginosa*
Genetic reagent (plasmid)	pJHA-Gn-ornVc H158A	This study		H158A from pDONR cloned into pJHA for expressing in *P. aeruginosa*
Antibody	Mouse anti-HA monoclonal antibody	Sigma; catalog number H9658		1:5000
Antibody	Goat Anti-Mouse IgG-HRP conjugate	Sigma; catalog number A9917		1:5000
DNA fragment	5'-GGATCCATGGCAGCCGGTGAAAG CATGGCACAGCGTATGGTTTGGGTT GATCTGGAAATGACCGGTCTGGATA TTGAAAAAGATCAGATTATTGAAATG GCCTGCCTGATTACCGATAGCGATCT GAATATTCTGGCAGAAGGTCCGAAT CTGATTATCAAACAGCCGGATGAACT GCTGGATAGCATGAGCGATTGGTGT AAAGAACATCATGGTAAAAGCGGTCT GACCAAAGCAGTTAAAGAAAGCACCA TTACACTGCAGCAGGCCGAATATGAAT TTCTGAGCTTTGTTCGTCAGCAGACC CCTCCGGGTCTGTGTCCGCTGGCAGG TAATAGCGTTCATGAAGATAAAAAGTT TCTGGATAAGTATATGCCGCAGTTTAT GAAGCATCTGCATTATCGCATTATTGAT GTGAGCACCGTTAAAGAACTGTGTCGT CGTTGGTATCCGGAAGAATATGAGTTT GCACCGAAAAAAGCAGCAAGCCATCGT GCACTGGATGATATTAGCGAAAGCATC AAAGAGCTGCAGTTTTATCGCAACAAC ATCTTCAAAAAGAAAATCGACGAGAAAA AACGCAAAATCATCGAAAACGGCGAAAA CGAAAAAACCGTTAGCTAAGCGGCCGC-3'	GeneArt		custom DNA fragment for REXO2 that is codon-optimized for *E. coli*
DNA fragment	5'-GGATCCATGAGCTTTAGCGATCAGAAT CTGATTTGGATTGATCTGGAAATGACCG GTCTGGACCCGGAAATGCATAAAATCAT TGAAATGGCAACCATCGTGACCGATAGCG AACTGAATATTCTGGCAGAAGGTCCGGTT ATTGCAATTCATCAGCCGGAAAGCGAACT GGCAAAAATGGATGAATGGTGTACCACCA CCCATACCGCAAGCGGTCTGGTTGCACGT GTTCGTCAGAGCCAGGTTAGCGAAGAAGA AGCAATTGATCAGACCCTGGCATTTCTGAA ACAGTGGGTTCCGGAAGGTAAAAGCCCGAT TTGTGGTAATAGCATTGGTCAGGATCGTCG CTTTCTGTATAAACATATGCCTCGTCTGGAA GCCTATTTCCATTATCGTTATATTGATGTGAG CACCATCAAAGAACTGACCCGTCGTTGGCAG CCGGAAGTTCTGAAAGAATTTAGCAAAACCG GTAGCCATCTGGCACTGGATGATATTCGTGA AAGCATTGCAGAGCTGCAGTTTTATCGTAAA GCCGTGTTTAAAATCTAAGCGGCCGC-3'	GeneArt		custom DNA fragment for OrnVc that is codon-optimized for *E. coli*
RNA primer	5'-GG-3'	Sigma		
RNA primer	5'-AGG-3'	Sigma		
RNA primer	5'-AAGG-3'	Sigma		
RNA primer	5'-AAAGG-3'	Sigma		
RNA primer	5'-AAAAGG-3'	Sigma		
RNA primer	5'-AAAAAGG-3'	Sigma		
RNA primer	5'-pGG-3'	Biolog; catalog number P023-01		
RNA primer	5'-pAA-3'	Biolog; catalog number P033-01		
RNA primer	5'-pAG-3'	GE Healthcare Dharmacon		
RNA primer	5'-pGA-3'	GE Healthcare Dharmacon		
RNA primer	5'-pGC-3'	GE Healthcare Dharmacon		
RNA primer	5'-pCG-3'	GE Healthcare Dharmacon		
RNA primer	5'-pCU-3'	GE Healthcare Dharmacon		
Software	Prism	GraphPad		
Software	XDS	Kabsch, 2010; PMID: 20124693		Distributed through SBGrid
Software	Pointless	Evans, 2006; PMID: 16369096		Distributed through SBGrid
Software	Scala	Evans, 2006; PMID: 16369096		Distributed through SBGrid
Software	Phenix	Adams et al., 2010; PMID: 20124702		Distributed through SBGrid
Software	Coot	Emsley et al., 2010; PMID: 20383002		Distributed through SBGrid
Software	Pymol	Schrödinger		
Software	Fujifilm Multi Gauge software v3.0	Fujifilm		

### Cloning, protein expression and purification

*orn* genes from *Vibrio cholerae* O1 El Tor VC0341 (residues 1–181) and *Homo sapiens* REXO2 (residues 33–237) were synthesized by Geneart (Life Technologies). Genes were cloned by ligation between BamHI and NotI sites of a modified pET28a vector (Novagen) yielding N-terminally His_6_-tagged small ubiquitin-like modifier (SUMO) fusion proteins cleavable by recombinant Ulp-1 protease.

Orn proteins were overexpressed in *E. coli* BL21 T7 Express cells (New England Biolabs). Fresh transformants were grown in Terrific Broth (TB) supplemented with 50 ug/mL kanamycin at 37°C to an OD_600_ ~1.0, at which point the temperature was reduced to 18°C and expression was induced by addition of 0.5 mM IPTG. Cells were harvested after 16 hr of expression by centrifugation and resuspended in a minimal volume of Ni-NTA binding buffer (25 mM Tris-Cl, 500 mM NaCl, 20 mM imidazole, pH 8.5) followed by flash freezing in liquid nitrogen.

Cells were thawed and lysed by sonication. Cell debris was removed by centrifugation and clarified soluble lysate was incubated with Ni-NTA resin (Qiagen) pre-equilibrated with Ni-NTA binding buffer. Following one hour of binding, the resin was washed three times with 10 column volumes of Ni-NTA binding buffer, and then eluted with six column volumes of Ni-NTA elution buffer (25 mM Tris-Cl, 500 mM NaCl, 350 mM imidazole, pH 8.5). Eluates were buffer exchanged into gel filtration buffer (25 mM Tris-Cl, 150 mM NaCl, pH 7.5) via a HiPrep 26/10 desalting column (GE Healthcare), followed by overnight incubation with Ulp-1 to cleave off the His_6_-tagged SUMO moiety. Untagged Orn proteins were recovered in the flow-through of a HisTrap Ni-NTA column (GE Healthcare), separated from His_6_-SUMO, uncleaved proteins and His_6_-tagged Ulp-1. Orn was concentrated via Amicon Ultra 10K concentrator prior to loading onto a HiLoad 16/60 Superdex 200 gel filtration column (GE Healthcare) equilibrated in gel filtration buffer. Fractions containing Orn were pooled and concentrated to 100 mg/mL, frozen in liquid nitrogen, and stored at −80°C.

### Bacterial strains, plasmids and growth conditions

Strains and plasmids are listed in Key Resources table. *P. aeruginosa* is routinely grown in LB supplemented with the appropriate antibiotic (50 µg/mL carbenicillin or 75 µg/mL gentamicin) at 37°C.

### Protein crystallography

REXO2-RNA and Orn*_Vc_*-RNA complexes (pGpG and pApA from BioLog Life Science Institute, other nucleotides from GE Healthcare Dharmacon) were formed prior to crystallization by mixing a 1:2 molar ratio of Orn:RNA in gel filtration buffer, followed by incubation for 30 min at the crystallization temperature. Orn-RNA complexes (10–30 mg/ml) were crystallized via hanging-drop vapor diffusion by mixing equal volumes (0.8 μl) of sample with reservoir solution. Orn*_Vc_* crystals grew at 20°C over a reservoir solution that was composed of 0.1 M BisTris (pH 5.5), 17% polyethylene glycol 3350, and 20% xylitol. Crystals were flash-frozen in liquid nitrogen. REXO2 crystals grew at 4°C, using a reservoir comprised of 0.2 M sodium malonate (pH 5.5) and 15–20% polyethylene glycol 3350. REXO2 crystals were soaked in cryoprotectant of reservoir solution supplemented with 25% glycerol prior to flash freezing with liquid nitrogen. All crystals were stored in liquid nitrogen. Data were collected by synchrotron radiation at 0.977 Å on frozen crystals at 100 K at beamline F1 of the Cornell High Energy Synchrotron Source (CHESS). Diffraction data sets were processed using XDS, Pointless, and Scala (Evans, 2006; Kabsch, 2010). The initial structures were solved by Molecular Replacement using the software package Phenix (Adams et al., 2010) and the unpublished coordinates of *E. coli* Orn (PDB: 2igi) as the search model. Manual model building and refinement were carried out with Coot (Emsley et al., 2010) and Phenix, respectively. Illustrations were prepared in Pymol (Version 2.2.0, Schrodinger, LLC). All software packages were accessed through SBGrid (Morin et al., 2013). Crystallographic statistics are shown in Figure 2—source data 1.

### Site-directed mutagenesis

To create the point mutants of *orn_Vc_*, mutations were generated by using the Q5 Site-Directed Mutagenesis Kit (New England Biolabs). All mutations were verified by sequencing.

### Western blot analysis

Overnight cultures were diluted to OD_600_ of ~0.02 in media with 0.2% arabinose with 75 µg/mL gentamicin. Culture density was monitored by OD_600_. At OD_600_ ~1.5, cells were collected by centrifugation and resuspended in 1/15 vol of 1x PBS. Proteins were immediately precipitated by a modified MeOH/CHCl_3_ procedure (sample/MeOH/CHCl_3_: 1/1/0.25 [Wessel and Flügge, 1984]). Samples were separated on 12% SDS-PAGE, transferred to PVDF membranes and blocked with Tris buffered saline (50 mM Tris, pH7.4, 200 mM NaCl) with 5% non-fat milk. HA-tags were detected using mouse anti-HA (Sigma H9658) and goat anti-mouse IgG-HRP conjugate (Sigma A9917). HRP signal was developed using chemiluminescence per manufacture protocol (Sigma).

### Labeling of RNAs

5’ un-phosphorylated RNAs were purchased from TriLink Biotechnologies or Sigma. Each RNA was subjected to radioactive end-labeling or non-radioactive phosphorylation by T4 Polynucleotide Kinase (New England Biolabs). Each RNA was subjected to phosphorylation with equimolar concentrations of either ^32^P-γ-ATP or ATP, T4 PNK, and 1X T4 PNK Reaction Buffer. Reactions comprising a final concentration of either 0.5 µM radiolabeled RNA or 2.0 µM phosphorylated RNA were incubated at 37°C for 40 min, followed by heat inactivation of T4 PNK at 65°C for 20 min.

### Protein expression and purification for biochemical assays

*E. coli* T7Iq strains harboring expression vector pVL847 expressing an His_10_-MBP-Orn and His_10_-MBP-Orn mutants from *V. cholerae* were grown overnight, subcultured in LB M9 fresh media supplemented with 15 μg/ml gentamicin and grown to approximately OD_600_0.5 ~ 1.0 at 30°C. Expression was induced with 1 mM IPTG for 4 hr. Induced bacteria were collected by centrifugation and resuspended in 10 mM Tris, pH 8, 100 mM NaCl, and 25 mM imidazole. After addition of 10 µg/mL DNase, 25 µg/mL lysozyme, and 1 mM PMSF, bacteria were lysed by sonication. Insoluble material was removed by centrifugation. The His-fusion protein was purified by separation over a Ni-NTA column. Purified proteins were pooled and dialyzed for 1 hr and overnight against 10 mM Tris, pH 8, 100 mM NaCl. The proteins were dialyzed for 3 hr in 10 mM Tris, pH 8, 100 mM NaCl, and 50% (vol/vol) glycerol, aliquoted, and flash frozen with liquid nitrogen and stored at −80° ˚C.

### Size-exclusion chromatography coupled multiangle light scattering (SEC-MALS)

5 mg/ml (0.24 mM) of purified protein (wild-type Orn*_Vc_*, Orn*_Vc_*-D^12^A, or Orn*_Vc_*-R^130^A) was injected onto a Superdex 200 Increase 10/300 column (GE Healthcare) equilibrated with gel filtration buffer (25 mM Tris-Cl, pH 7.5, 150 mM NaCl). Samples were run continuously at a flow rate of 0.75 ml/min through the gel filtration column coupled to a static 18-angle light scattering detector (DAWN HELIOS-II) and a refractive index detector (Optilab T-rEX), with data being collected every second. Data analysis was performed with ASTRA VI, yielding the molar mass and mass distribution (polydispersity) of the sample, using monomeric BSA (Sigma; 5 mg/ml) to normalize the light scattering detectors and as a control sample.

### Preparation of whole cell lysates

Overnight cultures of *P. aeruginosa* PA14 WT, *Δorn* mutant, or complemented strains were subcultured into fresh media with antibiotic and 1 mM IPTG, grown at 37°C with shaking to OD_600_ ~ 2.5. All bacteria samples were collected by centrifuge and resuspended in 1/10 vol of 100 mM NaCl, and 100 mM Tris, pH 8, also supplemented with 25 µg/mL lysozyme, 10 µg/mL DNase, and 1 mM PMSF and stored at −80°C.

### Oligoribonuclease cleavage reactions

Phosphorylated RNA (1.0 µM), including trace amounts of radiolabeled substrate, was subjected to cleavage by either 5.0 nM or 1.0 µM purified Orn at room temperature. These reactions were in the presence of 10 mM Tris, pH 8.0, 100 mM NaCl, and 5 mM MgCl_2_. At the appropriate times, aliquots of the reaction were removed and quenched in the presence of 150 mM EDTA on ice and heat inactivated at 95°C for 5 min. Activity of whole cell lysates against ^32^P-labeled oligoribonucleotide substrates was performed at room temperature in reaction buffer (10 mM Tris, pH 8, 100 mM NaCl, and 5 mM MgCl_2_). At the indicated times, the reaction was stopped by the addition of 0.2 M EDTA and heated at 98°C for 5 min. Samples were separated on denaturing 20% PAGE containing 1x TBE and 4 M urea. The gels were imaged using Fujifilm FLA-7000 phosphorimager (GE) and analyzed for the appearance of truncated ^32^P-labeled products. The intensity of the radiolabeled nucleotides was quantified using Fujifilm Multi Gauge software v3.0.

### DRaCALA measurement of dissociation constants

To measure *K_d_*, serial dilutions of purified His_10_-MBP-Orn, His_10_-MBP Orn mutants, or untagged Orn in binding buffer (10 mM Tris, pH 8, 100 mM NaCl, and 5 mM CaCl_2_) were mixed with radiolabeled nucleotides, applied to nitrocellulose sheets, dried, imaged and *K_d_* values were calculated as described previously (Roelofs et al., 2011; Patel et al., 2014).

### Aggregation assay

A colony of each strain of *P. aeruginosa* grown in LB agar plates supplemented with 50 µg/mL carbenicillin was inoculated into borosilicate glass tubes containing 2.5 mL of LB supplemented with 0.1 mM IPTG. The cultures were placed in a fly-wheel in 37°C incubator to spin for 18 ~ 22 hr. Culture tubes were allowed to settle at room temperature for 10 min and photographed.

### Colony morphology

Indicated strains are grown overnight at 37°C in LB agar with either 50 µg/mL of carbenicillin or 15 µg/mL of gentamicin, as appropriate. Three independent colonies are inoculated into LB with the appropriate antibiotics at 37°C with shaking. The bacteria were subcultured and grown until OD_600_ between 1.0 and 1.5. All cultures were diluted to ~10,000 CFU per mL. To observe colony size of multiple colonies of multiple strains, 10 µL of each strain were dripped in parallel on the same LB plates with the appropriate antibiotic and indicated IPTG conditions to ensure all strains were grown with the same media and conditions. For all strains tested, the control strains were always included on the same plate.

### Data deposition

The atomic coordinates and structure factors have been deposited in the Protein Data Bank, www.rcsb.org (PDB ID codes 6N6A, 6N6C, 6N6D, 6N6E, 6N6F, 6N6G, 6N6H, 6N6I, 6N6J, and 6N6K).

## Data Availability

The atomic coordinates and structure factors have been deposited in the Protein Data Bank, www.rcsb.org (PDB ID codes 6N6A, 6N6C, 6N6D, 6N6E, 6N6F, 6N6G, 6N6H, 6N6I, 6N6J, and 6N6K). Source data files have been provided for Figures. The following datasets were generated: LormandJD SondermannH 2019Vibrio cholerae Oligoribonuclease bound to pGGProtein Data Bank6N6A LormandJD SondermannH 2019Vibrio cholerae Oligoribonuclease bound to pAAProtein Data Bank6N6C LormandJD SondermannH 2019Vibrio cholerae Oligoribonuclease bound to pAGProtein Data Bank6N6D LormandJD SondermannH 2019Vibrio cholerae Oligoribonuclease bound to pGAProtein Data Bank6N6E LormandJD SondermannH 2019Vibrio cholerae Oligoribonuclease bound to pGCProtein Data Bank6N6F LormandJD SondermannH 2019Vibrio cholerae Oligoribonuclease bound to pCGProtein Data Bank6N6G LormandJD SondermannH 2019Vibrio cholerae Oligoribonuclease bound to pCpUProtein Data Bank6N6H LormandJD SondermannH 2019Human REXO2 bound to pGGProtein Data Bank6N6I LormandJD SondermannH 2019Human REXO2 bound to pAAProtein Data Bank6N6J LormandJD SondermannH 2019Human REXO2 bound to pAGProtein Data Bank6N6K

## Additional files

Supplementary file 1. Primers.

Transparent reporting form

## References

[bib1] Adams PD, Afonine PV, Bunkóczi G, Chen VB, Davis IW, Echols N, Headd JJ, Hung LW, Kapral GJ, Grosse-Kunstleve RW, McCoy AJ, Moriarty NW, Oeffner R, Read RJ, Richardson DC, Richardson JS, Terwilliger TC, Zwart PH (2010). PHENIX: a comprehensive Python-based system for macromolecular structure solution. Acta Crystallographica Section D Biological Crystallography.

[bib2] Baker NA, Sept D, Joseph S, Holst MJ, McCammon JA (2001). Electrostatics of nanosystems: application to microtubules and the ribosome. PNAS.

[bib3] Bandyra KJ, Luisi BF (2013). Licensing and due process in the turnover of bacterial RNA. RNA Biology.

[bib4] Baraquet C, Murakami K, Parsek MR, Harwood CS (2012). The FleQ protein from *pseudomonas aeruginosa* functions as both a repressor and an activator to control gene expression from the pel operon promoter in response to c-di-GMP. Nucleic Acids Research.

[bib5] Bradley DE (1974). The adsorption of Pseudomonas aeruginosa pilus-dependent bacteriophages to a host mutant with nonretractile pili. Virology.

[bib6] Bruni F, Gramegna P, Oliveira JM, Lightowlers RN, Chrzanowska-Lightowlers ZM (2013). REXO2 is an oligoribonuclease active in human mitochondria. PLOS ONE.

[bib7] Cheng ZF, Deutscher MP (2002). Purification and characterization of the *Escherichia coli* exoribonuclease RNase R. comparison with RNase II. The Journal of Biological Chemistry.

[bib8] Cheng ZF, Deutscher MP (2005). An important role for RNase R in mRNA decay. Molecular Cell.

[bib9] Chin KH, Yang CY, Chou CC, Wang AH, Chou SH (2006). The crystal structure of XC847 from *xanthomonas campestris*: a 3'-5' oligoribonuclease of DnaQ fold family with a novel opposingly shifted helix. Proteins: Structure, Function, and Bioinformatics.

[bib10] Chu LY, Agrawal S, Chen YP, Yang WZ, Yuan HS (2019). Structural insights into nanoRNA degradation by human Rexo2. RNA.

[bib11] Cohen D, Mechold U, Nevenzal H, Yarmiyhu Y, Randall TE, Bay DC, Rich JD, Parsek MR, Kaever V, Harrison JJ, Banin E (2015). Oligoribonuclease is a central feature of cyclic diguanylate signaling in *pseudomonas aeruginosa*. PNAS.

[bib12] Crooks GE, Hon G, Chandonia JM, Brenner SE (2004). WebLogo: a sequence logo generator. Genome Research.

[bib13] D'Argenio DA, Calfee MW, Rainey PB, Pesci EC (2002). Autolysis and autoaggregation in *pseudomonas aeruginosa* colony morphology mutants. Journal of Bacteriology.

[bib14] Datta AK, Niyogi K (1975). A novel oligoribonuclease of *Escherichia coli*. II. mechanism of action. The Journal of Biological Chemistry.

[bib15] Druzhinin SY, Tran NT, Skalenko KS, Goldman SR, Knoblauch JG, Dove SL, Nickels BE (2015). A conserved pattern of Primer-Dependent transcription initiation in *Escherichia coli* and *Vibrio cholerae* Revealed by 5' RNA-seq. PLOS Genetics.

[bib16] Emsley P, Lohkamp B, Scott WG, Cowtan K (2010). Features and development of coot. Acta Crystallographica. Section D, Biological Crystallography.

[bib17] Evans P (2006). Scaling and assessment of data quality. Acta Crystallographica Section D Biological Crystallography.

[bib18] Fang M, Zeisberg WM, Condon C, Ogryzko V, Danchin A, Mechold U (2009). Degradation of nanoRNA is performed by multiple redundant RNases in *Bacillus subtilis*. Nucleic Acids Research.

[bib19] Frazão C, McVey CE, Amblar M, Barbas A, Vonrhein C, Arraiano CM, Carrondo MA (2006). Unravelling the dynamics of RNA degradation by ribonuclease II and its RNA-bound complex. Nature.

[bib20] Fürste JP, Pansegrau W, Frank R, Blöcker H, Scholz P, Bagdasarian M, Lanka E (1986). Molecular cloning of the plasmid RP4 primase region in a multi-host-range tacP expression vector. Gene.

[bib21] Galperin MY, Natale DA, Aravind L, Koonin EV (1999). A specialized version of the HD hydrolase domain implicated in signal transduction. Journal of Molecular Microbiology and Biotechnology.

[bib22] Ghosh S, Deutscher MP (1999). Oligoribonuclease is an essential component of the mRNA decay pathway. PNAS.

[bib23] Goldman SR, Sharp JS, Vvedenskaya IO, Livny J, Dove SL, Nickels BE (2011). NanoRNAs prime transcription initiation in vivo. Molecular Cell.

[bib24] Hall CL, Lee VT (2018). Cyclic-di-GMP regulation of virulence in bacterial pathogens. Wiley Interdisciplinary Reviews: RNA.

[bib25] Hengge R (2009). Principles of c-di-GMP signalling in bacteria. Nature Reviews Microbiology.

[bib26] Hickman JW, Tifrea DF, Harwood CS (2005). A chemosensory system that regulates biofilm formation through modulation of cyclic diguanylate levels. PNAS.

[bib27] Hsiao YY, Yang CC, Lin CL, Lin JL, Duh Y, Yuan HS (2011). Structural basis for RNA trimming by RNase T in stable RNA 3'-end maturation. Nature Chemical Biology.

[bib28] Hsiao YY, Duh Y, Chen YP, Wang YT, Yuan HS (2012). How an exonuclease decides where to stop in trimming of nucleic acids: crystal structures of RNase T-product complexes. Nucleic Acids Research.

[bib29] Hui MP, Foley PL, Belasco JG (2014). Messenger RNA degradation in bacterial cells. Annual Review of Genetics.

[bib30] Jenal U, Reinders A, Lori C (2017). Cyclic di-GMP: second messenger extraordinaire. Nature Reviews Microbiology.

[bib31] Kabsch W (2010). Integration, scaling, space-group assignment and post-refinement. Acta Crystallographica Section D Biological Crystallography.

[bib32] Kamp HD, Patimalla-Dipali B, Lazinski DW, Wallace-Gadsden F, Camilli A (2013). Gene fitness landscapes of *Vibrio cholerae* at important stages of its life cycle. PLOS Pathogens.

[bib33] Korada SK, Johns TD, Smith CE, Jones ND, McCabe KA, Bell CE (2013). Crystal structures of *Escherichia coli* exonuclease I in complex with single-stranded DNA provide insights into the mechanism of processive digestion. Nucleic Acids Research.

[bib34] Krasteva PV, Sondermann H (2017). Versatile modes of cellular regulation via cyclic dinucleotides. Nature Chemical Biology.

[bib35] Kulasakara H, Lee V, Brencic A, Liberati N, Urbach J, Miyata S, Lee DG, Neely AN, Hyodo M, Hayakawa Y, Ausubel FM, Lory S (2006). Analysis of *pseudomonas aeruginosa* diguanylate cyclases and phosphodiesterases reveals a role for bis-(3'-5')-cyclic-GMP in virulence. PNAS.

[bib36] Kwok CK, Merrick CJ (2017). G-Quadruplexes: prediction, characterization, and biological application. Trends in Biotechnology.

[bib37] Lee VT, Matewish JM, Kessler JL, Hyodo M, Hayakawa Y, Lory S (2007). A cyclic-di-GMP receptor required for bacterial exopolysaccharide production. Molecular Microbiology.

[bib38] Lee CW, Park SH, Jeong CS, Cha SS, Park H, Lee JH (2019). Structural basis of small RNA hydrolysis by oligoribonuclease (CpsORN) from colwellia psychrerythraea strain 34H. Scientific Reports.

[bib39] Li Z, Deutscher MP (1996). Maturation pathways for *E. coli* tRNA precursors: a random multienzyme process in vivo. Cell.

[bib40] Liu MF, Cescau S, Mechold U, Wang J, Cohen D, Danchin A, Boulouis HJ, Biville F (2012). Identification of a novel nanoRNase in *bartonella*. Microbiology.

[bib41] Malone JG, Jaeger T, Spangler C, Ritz D, Spang A, Arrieumerlou C, Kaever V, Landmann R, Jenal U (2010). YfiBNR mediates cyclic di-GMP dependent small colony variant formation and persistence in *pseudomonas aeruginosa*. PLOS Pathogens.

[bib42] Matsuyama BY, Krasteva PV, Baraquet C, Harwood CS, Sondermann H, Navarro MV (2016). Mechanistic insights into c-di-GMP-dependent control of the biofilm regulator FleQ from *pseudomonas aeruginosa*. PNAS.

[bib43] Mechold U, Ogryzko V, Ngo S, Danchin A (2006). Oligoribonuclease is a common downstream target of lithium-induced pAp accumulation in *Escherichia coli* and human cells. Nucleic Acids Research.

[bib44] Mechold U, Fang G, Ngo S, Ogryzko V, Danchin A (2007). YtqI from *Bacillus subtilis* has both oligoribonuclease and pAp-phosphatase activity. Nucleic Acids Research.

[bib45] Morin A, Eisenbraun B, Key J, Sanschagrin PC, Timony MA, Ottaviano M, Sliz P (2013). Collaboration gets the most out of software. eLife.

[bib46] Niyogi SK, Datta AK (1975). A novel oligoribonuclease of *Escherichia coli*. I. isolation and properties. The Journal of Biological Chemistry.

[bib47] Orr MW, Donaldson GP, Severin GB, Wang J, Sintim HO, Waters CM, Lee VT (2015). Oligoribonuclease is the primary degradative enzyme for pGpG in *pseudomonas aeruginosa* that is required for cyclic-di-GMP turnover. PNAS.

[bib48] Palace SG, Proulx MK, Lu S, Baker RE, Goguen JD (2014). Genome-wide mutant fitness profiling identifies nutritional requirements for optimal growth of *Yersinia pestis* in deep tissue. mBio.

[bib49] Patel DK, Gebbie MP, Lee VT (2014). Assessing RNA interactions with proteins by DRaCALA. Methods in Enzymology.

[bib50] Postic G, Danchin A, Mechold U (2012). Characterization of NrnA homologs from *mycobacterium tuberculosis* and *Mycoplasma pneumoniae*. RNA.

[bib51] Rahme LG, Stevens EJ, Wolfort SF, Shao J, Tompkins RG, Ausubel FM (1995). Common virulence factors for bacterial pathogenicity in plants and animals. Science.

[bib52] Roelofs KG, Wang J, Sintim HO, Lee VT (2011). Differential radial capillary action of ligand assay for high-throughput detection of protein-metabolite interactions. PNAS.

[bib53] Rolfs A, Montor WR, Yoon SS, Hu Y, Bhullar B, Kelley F, McCarron S, Jepson DA, Shen B, Taycher E, Mohr SE, Zuo D, Williamson J, Mekalanos J, LaBaer J (2008). Production and sequence validation of a complete full length ORF collection for the pathogenic bacterium *Vibrio cholerae*. PNAS.

[bib54] Römling U, Galperin MY, Gomelsky M (2013). Cyclic di-GMP: the first 25 years of a universal bacterial second messenger. Microbiology and Molecular Biology Reviews.

[bib55] Ross P, Weinhouse H, Aloni Y, Michaeli D, Weinberger-Ohana P, Mayer R, Braun S, de Vroom E, van der Marel GA, van Boom JH, Benziman M (1987). Regulation of cellulose synthesis in *acetobacter xylinum* by cyclic diguanylic acid. Nature.

[bib56] Sievers F, Wilm A, Dineen D, Gibson TJ, Karplus K, Li W, Lopez R, McWilliam H, Remmert M, Söding J, Thompson JD, Higgins DG (2011). Fast, scalable generation of high-quality protein multiple sequence alignments using clustal omega. Molecular Systems Biology.

[bib57] Stevens A, Niyogi SK (1967). Hydrolysis of oligoribonucleotides by an enzyme fraction from *Escherichia coli*. Biochemical and Biophysical Research Communications.

[bib58] Tal R, Wong HC, Calhoon R, Gelfand D, Fear AL, Volman G, Mayer R, Ross P, Amikam D, Weinhouse H, Cohen A, Sapir S, Ohana P, Benziman M (1998). Three cdg operons control cellular turnover of cyclic di-GMP in *acetobacter xylinum*: genetic organization and occurrence of conserved domains in isoenzymes. Journal of Bacteriology.

[bib59] The UniProt Consortium (2017). UniProt: the universal protein knowledgebase. Nucleic Acids Research.

[bib60] Ueda A, Wood TK (2009). Connecting quorum sensing, c-di-GMP, pel polysaccharide, and biofilm formation in *pseudomonas aeruginosa* through tyrosine phosphatase TpbA (PA3885). PLOS Pathogens.

[bib61] Vvedenskaya IO, Sharp JS, Goldman SR, Kanabar PN, Livny J, Dove SL, Nickels BE (2012). Growth phase-dependent control of transcription start site selection and gene expression by nanoRNAs. Genes & Development.

[bib62] Wessel D, Flügge UI (1984). A method for the quantitative recovery of protein in dilute solution in the presence of detergents and lipids. Analytical Biochemistry.

[bib63] Yu D, Deutscher MP (1995). Oligoribonuclease is distinct from the other known exoribonucleases of *Escherichia coli*. Journal of Bacteriology.

[bib64] Zuo Y, Deutscher MP (2002). The physiological role of RNase T can be explained by its unusual substrate specificity. Journal of Biological Chemistry.

